# Cynomolgus macaques as a translational model of human immune responses to yellow fever 17D vaccination

**DOI:** 10.1128/jvi.01516-23

**Published:** 2024-04-03

**Authors:** Nathalie Mantel, Fabienne Piras-Douce, Emilie Chautard, Ernesto Marcos-Lopez, Caroline L. Bodinham, Antonio Cosma, Virginie Courtois, Nina Dhooge, Sylviane Gautheron, Stefan H. E. Kaufmann, Kathleen Pizzoferro, David J. M. Lewis, Frédéric Martinon, Anke Pagnon, Franck Raynal, Nathalie Dereuddre-Bosquet, Roger Le Grand

**Affiliations:** 1 Research and Development, Sanofi, Marcy L'Etoile, France; 2 Université Paris-Saclay, INSERM, CEA, Center for Immunology of Viral, Auto-immune, Hematological and Bacterial diseases (IMVA-HB/IDMIT), Fontenay aux Roses, France; 3 Surrey Clinical Research Centre, University of Surrey, Guildford, Surrey, United Kingdom; 4 Max Planck Institute for Infection Biology, Berlin, Germany; Max Planck Institute for Multidisciplinary Sciences, Göttingen, Germany; 5 Hagler Institute for Advanced Study, Texas A&M University, College Station, Texas, USA; University of North Carolina at Chapel Hill, Chapel Hill, North Carolina, USA

**Keywords:** non-human primate, human, yellow fever, vaccine

## Abstract

The non-human primate (NHP) model (specifically rhesus and cynomolgus macaques) has facilitated our understanding of the pathogenic mechanisms of yellow fever (YF) disease and allowed the evaluation of the safety and efficacy of YF-17D vaccines. However, the accuracy of this model in mimicking vaccine-induced immunity in humans remains to be fully determined. We used a systems biology approach to compare hematological, biochemical, transcriptomic, and innate and antibody-mediated immune responses in cynomolgus macaques and human participants following YF-17D vaccination. Immune response progression in cynomolgus macaques followed a similar course as in adult humans but with a slightly earlier onset. Yellow fever virus neutralizing antibody responses occurred earlier in cynomolgus macaques [by Day 7[(D7)], but titers > 10 were reached in both species by D14 post-vaccination and were not significantly different by D28 [plaque reduction neutralization assay (PRNT)_50_ titers 3.6 Log vs 3.5 Log in cynomolgus macaques and human participants, respectively; *P* = 0.821]. Changes in neutrophils, NK cells, monocytes, and T- and B-cell frequencies were higher in cynomolgus macaques and persisted for 4 weeks versus less than 2 weeks in humans. Low levels of systemic inflammatory cytokines (IL-1RA, IL-8, MIP-1α, IP-10, MCP-1, or VEGF) were detected in either or both species but with no or only slight changes versus baseline. Similar changes in gene expression profiles were elicited in both species. These included enriched and up-regulated type I IFN-associated viral sensing, antiviral innate response, and dendritic cell activation pathways D3–D7 post-vaccination in both species. Hematological and blood biochemical parameters remained relatively unchanged versus baseline in both species. Low-level YF-17D viremia (RNAemia) was transiently detected in some cynomolgus macaques [28% (5/18)] but generally absent in humans [except one participant (5%; 1/20)].

**IMPORTANCE:**

Cynomolgus macaques were confirmed as a valid surrogate model for replicating YF-17D vaccine-induced responses in humans and suggest a key role for type I IFN.

## INTRODUCTION

The yellow fever virus (YFV), which belongs to the genus *Flavivirus* (family *Flaviviridae*), is the causative agent of the zoonotic disease yellow fever (YF); the disease is endemic to the tropics of Africa and South America (1, 2). The virus circulates predominantly in the sylvatic (or forest) cycle involving mosquitoes and non-human primates (NHPs), but human intrusion into this cycle can result in the transfer of the virus *via* mosquitoes from NHPs to humans and, subsequently, between humans (urban cycle). A wide range of NHPs have been implicated in the sylvatic cycle including monkeys from the genera *Alouatta* (howler monkeys)*, Aotus* (night/owl monkey)*, Ateles* (spider monkey)*, Callithrix* (marmosets)*, Cebus* (capuchin monkeys)*, Leontopithecus* (lion tamarins)*, Sapajus* (robust capuchin monkeys) and *Saimiri* (squirrel monkeys) in the Americas and *Papio* (baboons)*, Colobus* (colobus monkeys)*, Cercopithecus* (vervet monkeys)*, Cercocebus* (mangabeys)*, Pan troglodytes* (chimpanzees), and *Galago* (bush babies) in Africa (3–5). Most human and NHP YFV infections (>55%) are asymptomatic (6). When present, clinical symptoms are broad, ranging from mild self-limiting nonspecific febrile illness, which deteriorates to severe toxic multisystem disease in 15% of cases (2, 7), with 20%–50% case fatality. There are an estimated 200,000 yellow fever cases annually worldwide resulting in 30,000 deaths (though 78,000 deaths were estimated to have occurred in Africa in 2013 (8)), with Africa bearing 90% of the global burden (2).

The live-attenuated YF-17D vaccine substrain was first developed in the 1930s, and which is still widely used today in the manufacture of YF vaccines, provides highly effective protective immunity (1). The YF-17D vaccine strain was derived from the wild-type YFV Asibi strain after serial passages in chicken and mouse embryo tissue, though the mechanism of attenuation and the determinants of immunogenicity remain poorly understood (9, 10). Nonetheless, YF-17D-induced neutralizing antibodies are a predictive proxy for protection against wild‐type YFV (11), are detectable within 10 days after vaccination (11), and last for more than 30 years (12, 13). Thus, a single dose of YF-17D vaccine may confer life-long protective immunity against YF disease (2).

Although the YF-17D vaccine has a good safety profile, serious adverse events involving vaccine-associated viscerotropic and neurotropic manifestations, albeit at very low frequency, have been reported (14, 15). Nonetheless, due to its well-established overall safety profile and effectiveness, the YF-17D genetic backbone has been used to create several vaccine candidates and continues to be developed as an antigen delivery system for other flaviviruses or pathogens. Despite the ongoing use and further development of the YF-17D vaccine, the mechanisms of protection and those underlying adverse events are incompletely understood, nor are the YFV-human interactions that cause disease. There are scientific and ethical limits to the types of studies that can be undertaken in humans, in particular when considering the comparison of cellular and molecular changes following the administration of live-attenuated vaccines versus wild-type infections, or associated blood biomarker profiles with innate and adaptive responses in inductive tissues, lymphoid organs, and effector sites. The use of animal models is unavoidable and so far cannot be replaced by alternative *in vitro* approaches because the complex host-virus interactions to be elucidated occur at the whole body level.

NHPs are probably the most relevant models for human YF disease due to their close relationship, natural susceptibility to YFV infection, and similarities in response to infection and pathogenesis (16). Furthermore, the WHO suggests that NHPs (specifically, rhesus and cynomolgus macaques) should be used for the preclinical safety and immunogenicity characterization of new YF vaccine candidates, including for the evaluation of neurotropism and viscerotropism (17). However, the extended characterization of vaccine interactions with host factors, and systemic and tissue mechanisms of vaccine-induced long-term protective immunity, requires careful identification of similarities and dissimilarities between human and animal responses (18).

Virus-host interactions after immunization with YF-17D and the mechanisms inducing protective immune responses in NHPs (rhesus macaques) (19) and humans can be assessed using systems biology approaches (20–23). Most systems biology studies with YF-17D vaccines conducted so far aimed to generate large-scale data to identify innate signatures that would help predict long-term responses in humans (20–24). Only one systems biology study has been performed to date with the NHP model (rhesus macaques; natural range extends from Eastern China through to Western India and Pakistan (25)), but this had a limited sample size (*n* = 3) (19).

Here, we undertook the first direct head-to-head assessment of responses to YF-17D vaccination in humans and cynomolgus macaques [natural range extends from Indonesia, Philippines and through mainland southeast Asia (Indochina region), including Myanmar, Thailand, Cambodia, Vietnam, and Laos (25)], and evaluated the accuracy of the animal model for evaluation of YF vaccine immunogenicity and safety. We characterized and compared the innate and specific responses to the YF-17D vaccine and the transcriptome profiles in both species. Importantly, these studies suggest a key role for type I IFN in the early immune response to the YF-17D vaccine and the translatability of the cynomolgus macaque model to humans for studying mechanisms of protective immunity.

## RESULTS

### Adaptive response to YF-17D vaccination in humans and non-human primates

To allow for a combined analysis of responses to the YF-17D vaccine and a direct translational assessment that can better link observations between species, two series of parallel studies were performed in 20 adult (YF-naïve) human participants [age 21–42 years (mean age 26.5 years); previously described elsewhere (26)] and 18 young adult cynomolgus macaques [age 2.7–3.6 years (mean age 3.5 years); weight 3.9–5.3 kg (mean 4.4 kg)] with similar sampling times and analyses undertaken before and after vaccination with the YF-17D vaccine. The ethnicity of the human participants was as follows: Asian (*n* = 4), white (*n* = 13), and other (*n* = 3) (26).

**Fig 1:**
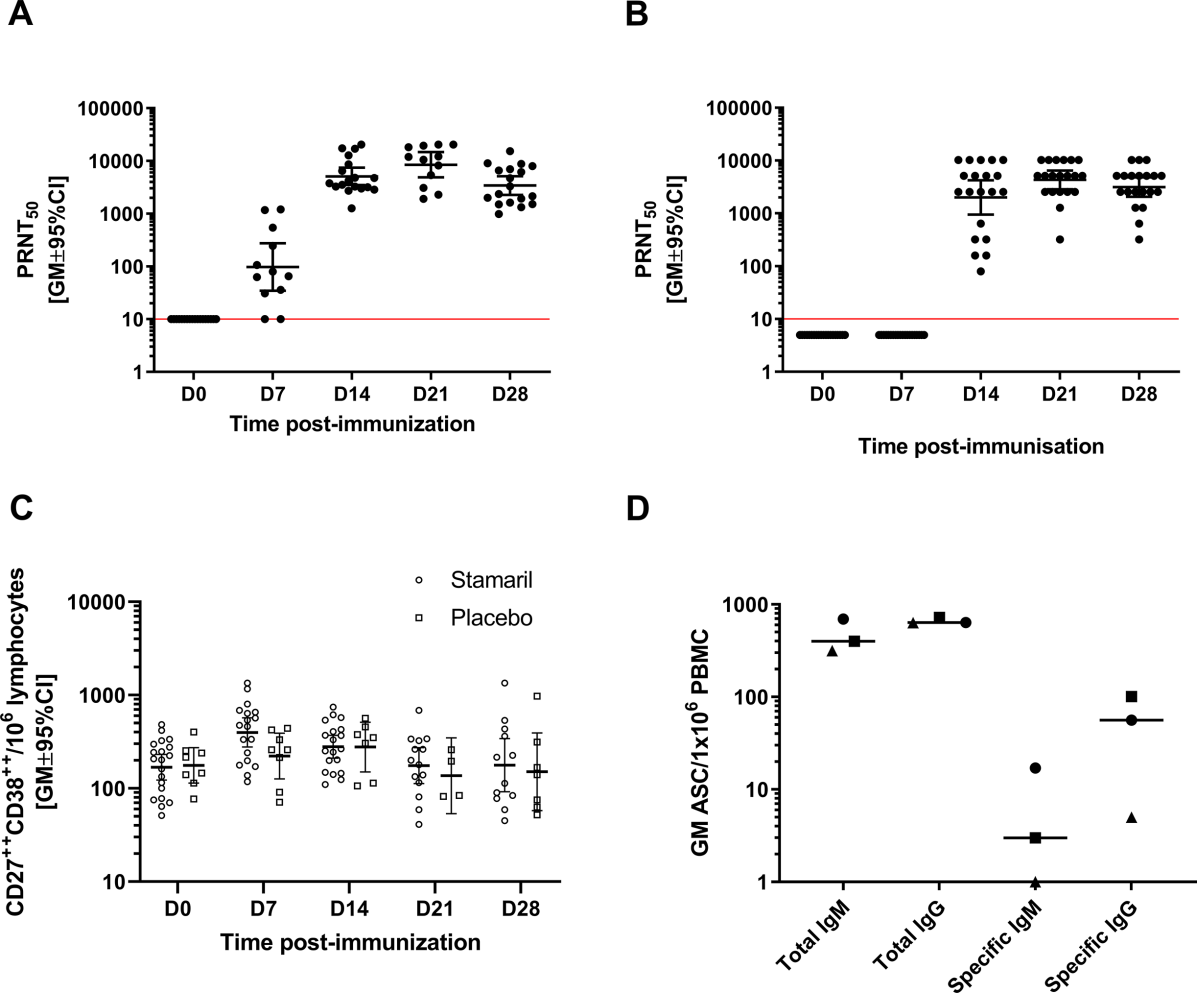
YFV-neutralizing antibody titers and induction of plasmablasts. Serum PRNT_50_ titers in (**A**) cynomolgus macaques (*n* = 18) and (**B**) humans (*n* = 20), (**C**) plasmablast kinetic profile analysis in humans by flow cytometry after staining with anti-CD19, CD3, CD20, CD27, and CD38 antibodies (Stamaril *n* = 20; Placebo *n* = 8), and (**D**) total and YF-17D-specific IgM and IgG secreting plasmablasts measured by FluoroSPOT in a subgroup of cynomolgus macaques (*n* = 3) at D7 post-vaccination. CI = confidence interval; GM = geometric mean; ASC = antibody-secreting cells; PBMC = peripheral blood mononuclear cells.

YFV-neutralizing antibodies, here evaluated using the 50% plaque reduction test [plaque reduction neutralization test (PRNT)_50_], are a recognized correlate of protection against YF disease. Thus, we first demonstrated that human participants (*n* = 20) and cynomolgus macaques (*n* = 18) injected subcutaneously in the deltoid (human) or upper back (cynomolgus macaques) region with the same dose of commercial YF-17D vaccine (Stamaril, Sanofi) develop similar YFV-neutralizing antibody profiles (Fig. 1A and B). Neutralizing antibody responses occurred earlier in cynomolgus macaques than in human participants; at Day 7 (D7), 80% (10/12) of cynomolgus macaques had seroconverted (neutralizing titers > 10 [1 Log]) [mean titer 1.9 Log (µPRNT_50_)] compared with none of the human participants. All cynomolgus macaques and human participants were YF naïve at baseline (seronegative), and all seroconverted (i.e., achieved YFV-neutralizing antibody titer >10) by Day 14 (D14). The mean neutralizing antibody titers in cynomolgus macaques and human participants were significantly different at D14 post-vaccination (3.7 Log vs 3.3 Log, respectively, *P* = 0.012) but not at D21 (*P* = 0.174) and D28 (*P* = 0.821) post-vaccination.

We analyzed the changes in frequencies of total plasmablasts in peripheral blood up to D28 following the administration of YF-17D or placebo in human participants. As expected, the frequency of CD3^−^CD19^+^CD20^low/–^CD27^high^CD38^high^ plasmablasts peaked by D7 post-vaccination, the only time point significantly higher than placebo (*P* = 0.03) (Fig. 1C). Due to blood sampling limitations for cynomolgus macaques, we assessed YF antigen-specific antibody-secreting cell frequencies at D7 only to complement the plasmablast analysis in humans. As measured by *ex vivo* FluoroSpot (Fig. 1D), in a subgroup of cynomolgus macaques (*n* = 3), the YF-specific IgM-secreting plasmablasts were detectable in two of three cynomolgus macaques, and the YF-specific IgG-secreting plasmablasts were detectable in all three. The early differences in neutralizing antibody kinetics between species may have been associated with the difference in the changes in plasmablast frequencies.

### Reactogenicity of the YF-17D vaccine in humans and non-human primates

The reactogenicity of the YF-17D vaccine in human participants has been described in part elsewhere (26); the most common adverse events were fever, injection site pain, and headache, which were mostly of mild intensity. No adverse effects on body temperature or body weight were observed in cynomolgus macaques (Fig. S1). Hematology (Fig. S2), plasma biochemical, and hepatic functional profiles (Fig. S3) remained unchanged after vaccination compared with baseline in both cynomolgus macaques and human participants. C-reactive protein values were transiently slightly increased at D7 post-vaccination [D0: GMT 0.5 mg/L, 95% CI (0.34;0.73); D7: GMT 1.4 mg/L, 95% CI (0.88; 2.04)] in human participants but remained below the range associated with clinically relevant inflammation (<10 mg/L) (26). No increases in C-reactive protein levels were observed in cynomolgus macaques [D0: 5.38 mg/L, 95% CI (5.0;5.73); and from D1 to D14 was 4.14 mg/L to 5.76 mg/L]. All values remained below ˂10 mg/L, except one NHP with a transient increase in C-reactive protein at D5 (30 mg/dl). Vaccination was associated with no or only mild reactogenicity in both species. In addition, no or low levels of systemic inflammatory cytokines, chemokines, and growth factors (Fig. S4) were detected in either species, confirming the low adverse host reaction to the YF-17D vaccine. More than 50% of soluble factors assessed were below the limit of quantification in both humans and cynomolgus macaques. Low levels of systemic inflammatory cytokines (IL-1RA, IL-8, MIP-1α, IP-10, MCP-1, or VEGF) were detected in either or both species, but with no or only slight differences observed after vaccination at the selected time points versus baseline. In particular, there was no significant IL-1RA response in both species, and IL-8 was only slightly increased in cynomolgus macaques (between 0.4 and 0.8 Log) but not in humans.

### *In vivo* YF-17D replication following vaccine injection

**Fig 2:**
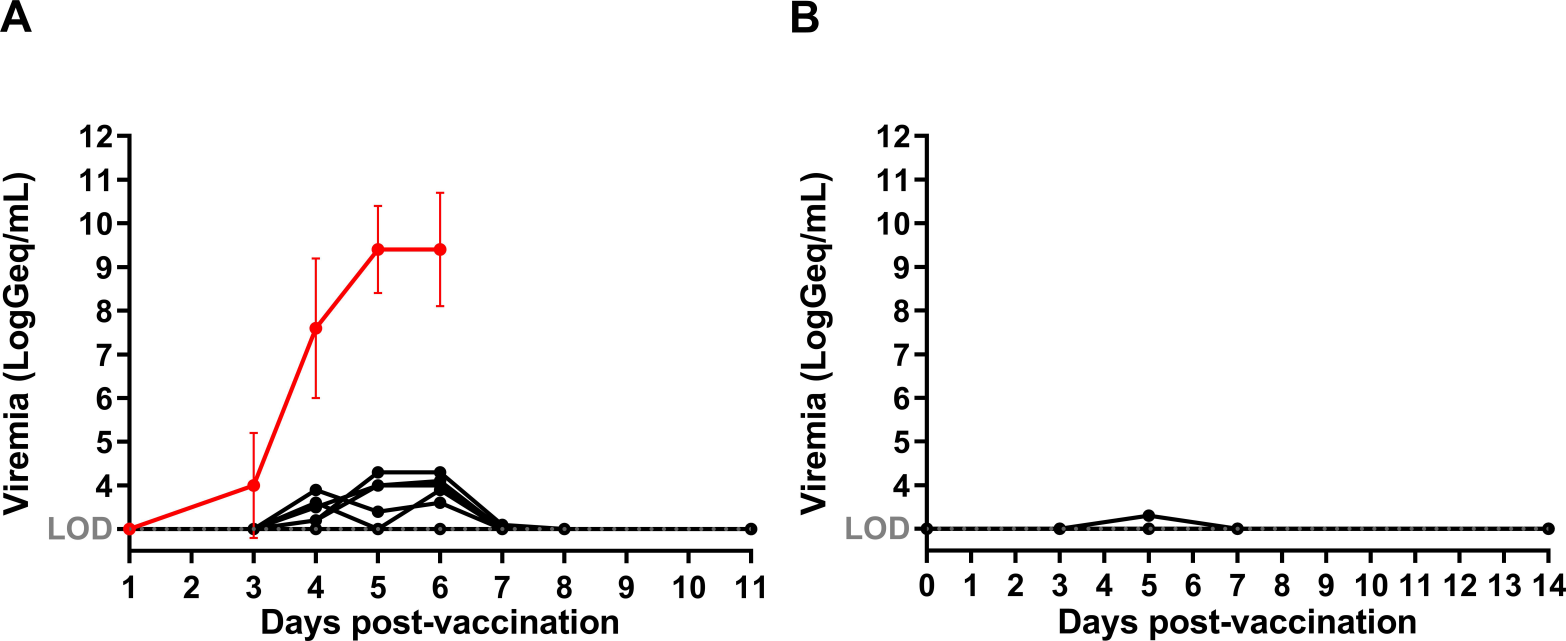
RNAemia detected by YF-NS5 qRT-PCR in individual (A) cynomolgus macaques (*N* = 18) and (B) humans (*N* = 20). Represented in red is the mean RNAemia concentration following infection with wild-type YFV Asibi strain in cynomolgus macaques (*N* = 15).

To assess the relationship between residual YF-17D replication and induced immunity, vaccine viremia was assessed by titration of viral RNA in plasma. Transient low vaccine RNAemia [<4.5 LogGeq/mL; corresponding to approximately 30 to 300 plaque-forming units (PFU)/mL] was detected by qRT-PCR in 28% (5/18) of cynomolgus macaques from D3 to D5 after vaccination, which lasted 1–3 days (Fig. 2A). RNAemia levels were at least 10,000-fold lower than those after wild-type infection in cynomolgus macaques (mean 9.4 ± 1.0 LogGeq/mL).

RNAemia was generally absent in humans [below the LOD (3.0 LogGeq/mL)], except for one participant (5%; 1/20) who had low-level RNAemia [below the LOQ (3.3 LogGeq/mL)] on D5 after vaccination (Fig. 2B). However, we detected antibodies against NS1 in both cynomolgus macaques and human participants indicative of viral replication (Fig. S5); NS1 is a non-structural protein produced during viral replication only. The NS1 protein content in the vaccine preparation was ≤0.2 ng/dose, thus unlikely to have directly induced the observed antibodies against NS1. Thus, YF-17D remains highly attenuated, with only very low viral replication observed or remaining undetectable in the plasma of most individuals in both species. The higher frequency of RNAemia detection in cynomolgus macaques may have contributed to the accelerated neutralizing antibody kinetics observed in this species.

### Vaccine-induced changes in myeloid and lymphoid leukocytes

Published data suggest a role for early changes in innate immune cells in shaping the long-term anti-YFV antibody response (27). We used mass cytometry to perform extensive characterization of differentiation and functional markers in myeloid and lymphoid cell populations in whole blood following vaccination with YF-17D. As we previously reported (18), many of the cell markers in cynomolgus macaques have human counterparts, emphasizing the relevance of this species as a model for evaluating human candidate vaccines. In addition, the antibody panel used in our study consisted of anti-human antibodies cross-reacting with macaque cell markers, thus facilitating the translatability of preclinical study protocols to human clinical trial samples.

**Fig 3:**
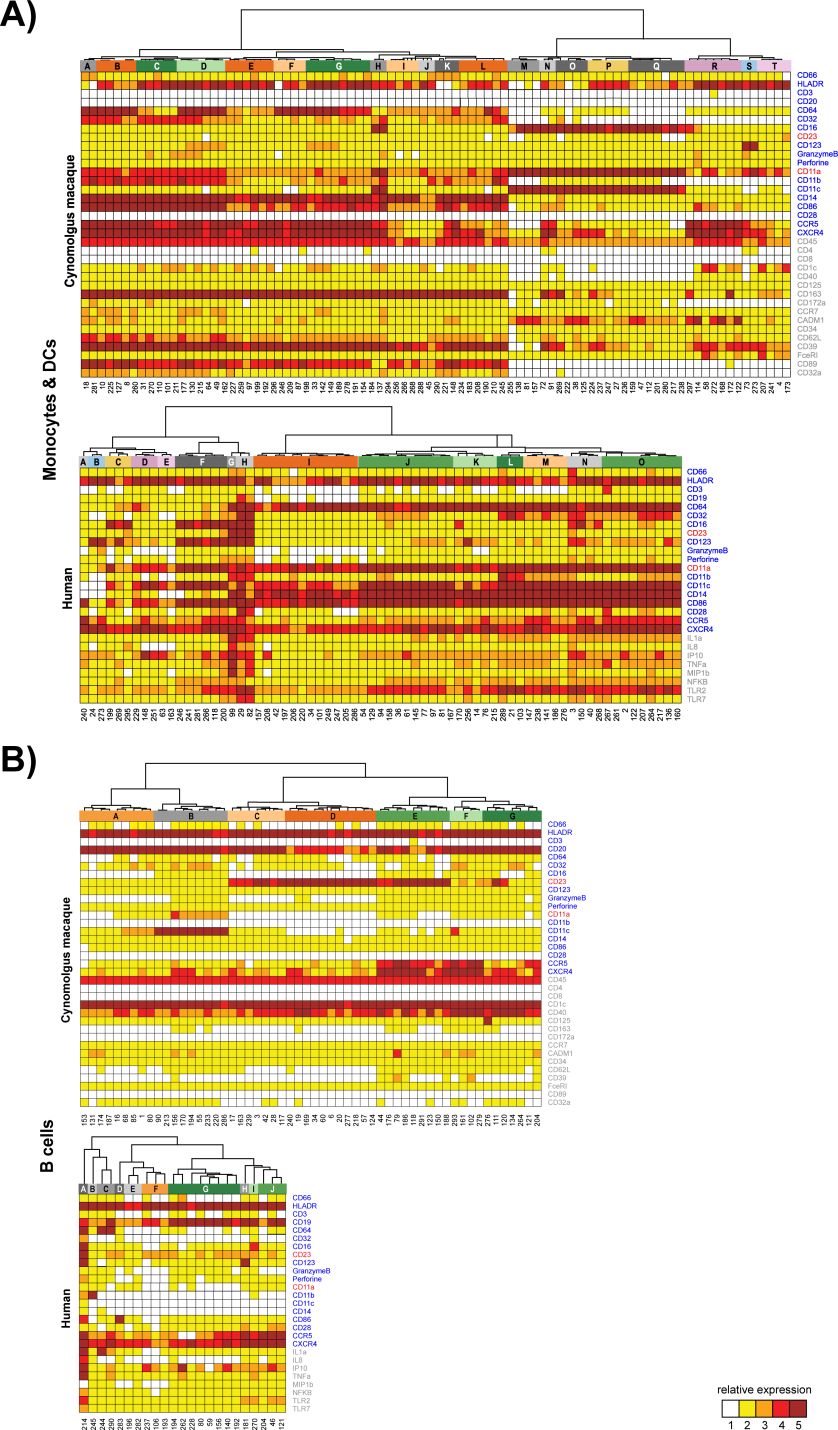
Phenotypic analysis of SPADE clusters. Hierarchical clustering was performed at the SPADE cluster level for (**A**) monocytes and DCs and (**B**) B cells. Marker intensities were uniformly divided into five categories based on the 2nd and 98th percentile range expression and represented in a five-tiered color scale from white to deep red. Based on the cluster dendrograms, groups of phenotypically similar clusters called phenotypic families were colored and defined by a capital letter.

**Fig 4:**
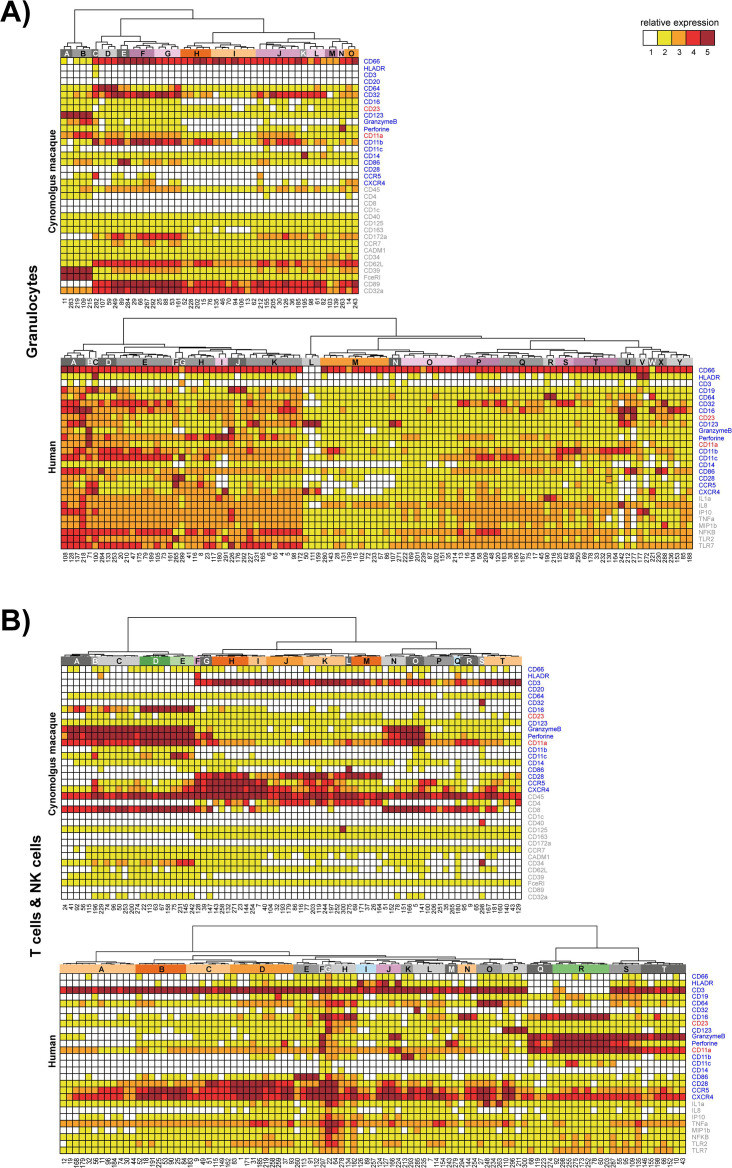
Phenotypic analysis of SPADE clusters. Hierarchical clustering was performed at the SPADE cluster level for (**A**) granulocytes and (**B**) T cells and NK cells. Marker intensities were uniformly divided into five categories based on the 2nd and 98th percentile range expression and represented in a five-tiered color scale from white to deep red. Based on the cluster dendrograms, groups of phenotypically similar clusters called phenotypic families were colored and defined by a capital letter.

A spanning-tree progression analysis of density-normalized events (SPADE) allowed for stratification into distinct cell populations including granulocytes, that is, neutrophils (CD66^+^CD11a^+^CD11b^+^) or basophils (CD66^–^HLA-DR^–^CD123^+^), monocytes (HLA-DR^+^CD14^+^), conventional dendritic cells (DCs) (HLA-DR^+^CD14^–^), plasmacytoid DCs (HLA-DR^+^CD123^+^), NK cells (CD3^-^GranzymeB^+^), T cells (CD3^+^), and B cells (CD19^+^ in humans or CD20^+^ in cynomolgus macaques). The SPADE clustering process organizes cells in a hierarchy of related phenotypes and places them into proximal or identical clusters. To visualize the expression profile of each of the markers by annotated SPADE clusters, a hierarchical clustering approach at the cluster level was performed (Fig. 3 and 4). Marker intensities were uniformly divided into five categories based on the 2nd and 98th percentile range expression. Additional descriptions of the phenotypic analysis of SPADE clusters are provided in the supplementary materials [see “Vaccine-induced changes in myeloid and lymphoid leukocyte (phenotypic analysis of SPADE clusters)” section].

**Fig 5:**
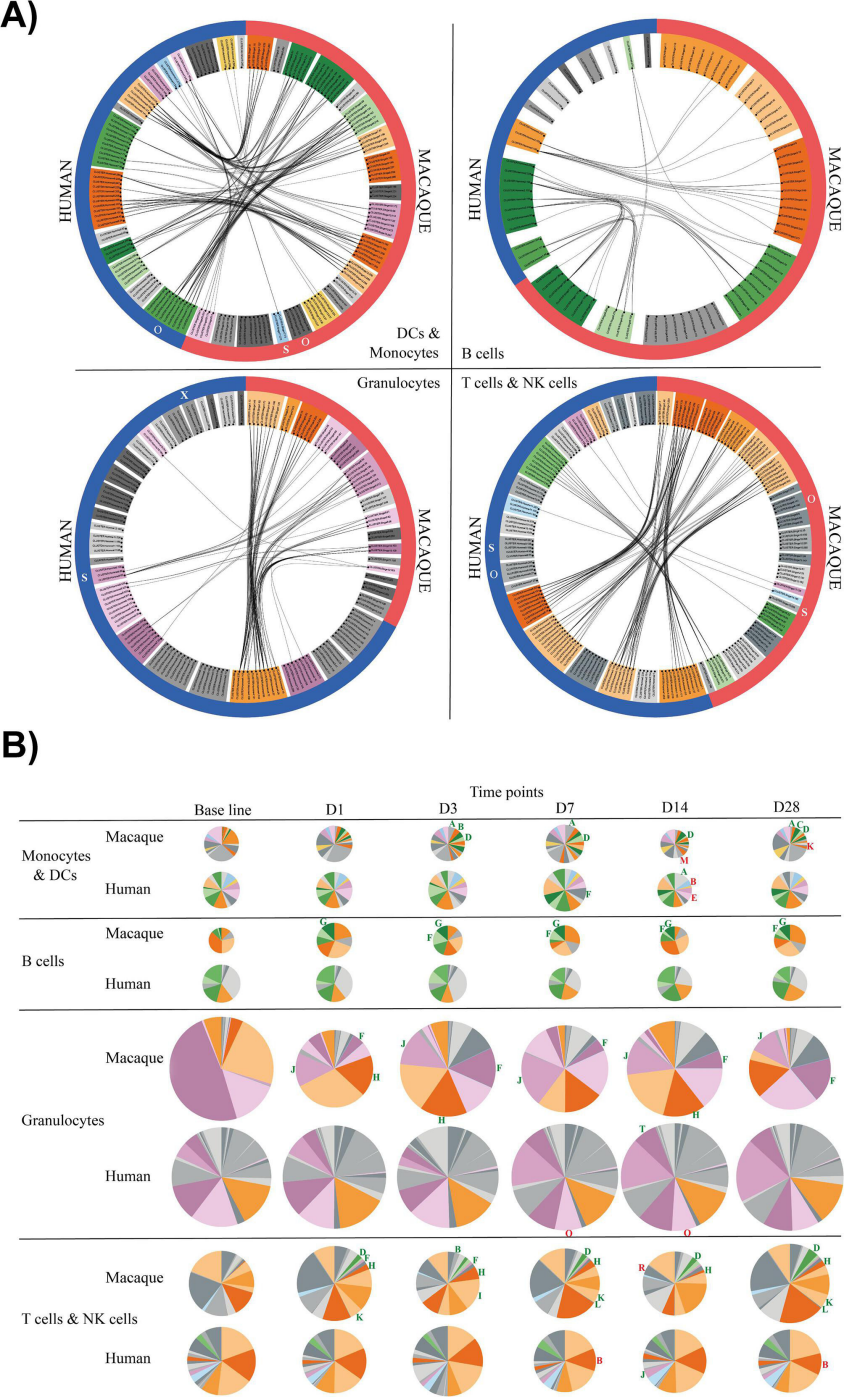
Phenotypic correlation of human and macaque clusters and phenotypic family abundance. (**A**) Circular graph representation showing the significant correlations between the SPADE clusters of the human and macaque analyses. Each node of the graph corresponds to a SPADE cluster and each line corresponds to a significant phenotype correlation between two human and macaque cell clusters. Phenotypically similar clusters are grouped into phenotypic families. Correlated families are colored in different shades of the same color. Phenotypic families colored in gray do not have any correlated clusters. (**B**) Phenotypic family abundance of monocytes and DCs, B cells, granulocytes, T cells, and NK cells compartments at all time points. The size of the pie chart is proportional to the cell concentration. Green and red capital letters mean significant enrichment or contraction of phenotypic families compared to the baseline. The color code for each phenotypic family is identical for the pie charts, the heatmaps, and the circular graphs.

Phenotypic similarities between human and cynomolgus macaque clusters were confirmed based on the Manhattan distances and are represented with different shades of the same color in Fig. 5A. We focused the comparative analysis on the large proportion of clusters with similar profiles of expressed markers in both species. To characterize changes in cell subpopulations induced by vaccination with YF-17D, a paired *t*-test corrected for multiple comparisons was performed to identify differentially enriched families (DEF) in both humans and cynomolgus macaques. DEFs were defined as phenotypic families that had undergone either a significant expansion or contraction relative to the total cells at baseline (D0), for any comparison at the selected five time points (*P* < 0.01, Fig. 5B).

The cynomolgus macaques responded earlier and with greater magnitude than humans, in agreement with observed YFV-neutralizing antibody kinetics. As shown in Fig. 5B, vaccination with YF-17D induced significant expansion of B cells, granulocytes, monocytes, NK, and T cells as early as D1 in cynomolgus macaques (see Table S1 for the *P*-values of the significant changes in the phenotypic families presented). Changes in human cells did not reach statistical significance before D7 and were of a lower magnitude than in cynomolgus macaques, additionally, the monocytes and neutrophils appeared less activated. In cynomolgus macaques, activated monocytes (HLA-DR^+^CD14^+^CD32, CD11a, and CD11b) expanded up to D28, while in humans two families of DCs, that is, E and F characterized by HLA-DR^+^CD14^–^CD11a, CD11b, and CD123^+/–^, respectively, were transiently expanded from D7 to D14 post-vaccination.

NK cells that highly expressed markers associated with cytotoxic function (stages D and B, and Granzyme B^high^, Perforin^high^) were elicited very early in cynomolgus macaques, by D1 and D3, following vaccination. However, because of the observed differences in T-cell kinetics between species, the early and transient changes (D1 to D3) that occurred in T-cell-activated populations, which migrate to lymphoid and effector tissues (HLA-DR^+^CD11a^+^CD28^+^CCR5^high^CXCR4^high^), were probably not captured in vaccinated humans (due to delayed baseline sampling before vaccination and/or the lower vaccine dose to weight ratio compared to cynomolgus macaques). The other cynomolgus macaque and human T-cell families appeared to display similar patterns of expansion and contraction phases between D7 and D28.

### Transcriptomic response to vaccination with YF-17D

The temporal gene expression pattern following vaccination with YF-17D in both cynomolgus macaques and human participants was assessed by comparing the transcript abundance in whole blood samples obtained at D1, D3, D7, D14, and D28 with their pre-vaccination baseline values (D–21).

**Fig 6:**
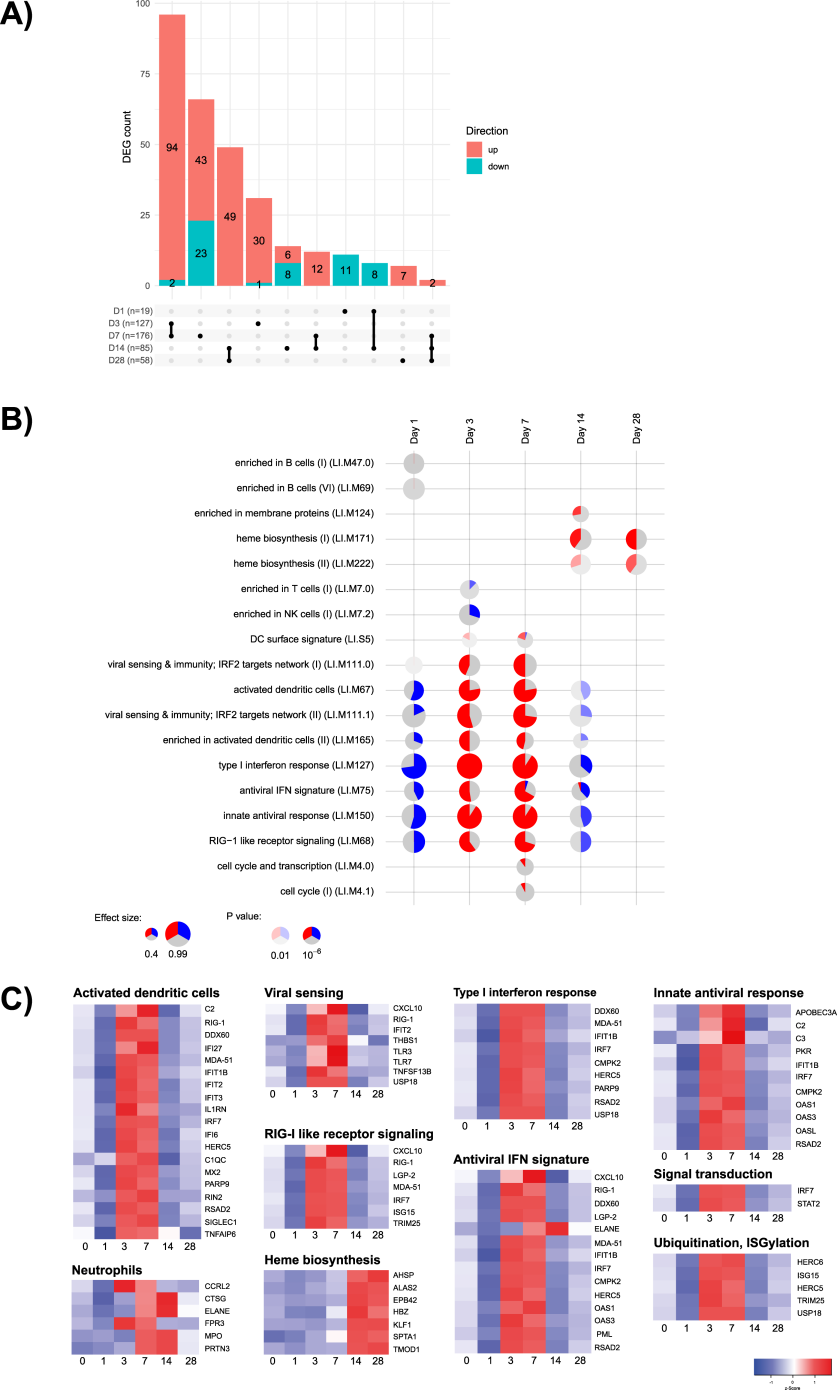
Functional characterization of cynomolgus macaque PBMCs transcriptomic response to vaccination with YF-17D up to D28 post-vaccination. (**A**) Upset plots illustrating the number of DEGs for each time point versus the baseline, and the number of genes in common for each comparison. Genes are considered in common if they have the same fold change direction. In the bar plot, red represents up-regulated and blue down-regulated genes. (**B**) Gene set enrichment analysis at each time point. The modules (in rows) are described by the titles followed by the original IDs. Each column corresponds to a different time point after vaccination with YF-17D. The size of each pie shows how strong the enrichment is and stronger colors indicate lower *P*-values. Red represents up-regulated and blue down-regulated genes. (**C**) Heat map of differentially expressed genes. The differentially expressed genes after vaccination with YF-17D were grouped into functional categories based on blood transcriptional modules. The mean expression value across replicates at each time is depicted in a red (high expression) to blue (low expression) color scheme.

In the first cohort of cynomolgus macaques (*n* = 12), 271 genes were significantly differentially expressed (over twofold decrease or increase) relative to baseline following vaccination with YF-17D on at least one time point (Fig. 6A). The peak occurrence of differentially expressed genes (DEGs) was at D3 (127 DEGs) and D7 (176 DEGs). As shown on the upset plot (Fig. 6A), the DEG at D3 and D7 are similar and most of which were up-regulated at these time points (124/127 DEGs at D3 and 151/176 DEGs at D7). This observation suggests a common activation involving the same gene regulatory networks at D3 and this activity continues at Day 7. Some of these genes had initially been down-regulated at D1 (19 DEGs), which could be explained by either recruitment of cells to distant organs, or by a baseline that evolved between D–21 (pre-baseline) and the injection day (D0). The number of genes was relatively low but strongly significant, and the kinetic of the response over several days is characteristic of live virus vaccines such as YF vaccines and is notably linked to the viral replication kinetics (26).

To decipher functional activities and pathways activated at D3 and D7, we applied gene set enrichment analysis using the blood transcriptional modules (BTMs, 346 at the time of the analysis) developed previously (28). These described aspects of the functioning immune system in humans and were used to infer potential biological functions in the changes in cynomolgus macaque transcript abundances. Three main and common sets of BTMs were enriched and up-regulated at D3 and D7 post-vaccination (Fig. 6B and C): viral sensing, antiviral innate response including interferon response, and DC activation modules.

The observations in the first cohort of cynomolgus macaques were confirmed in the second cohort (*n* = 6) on D3, D7, and D28 by comparing the transcript abundance in whole blood samples with their pre-vaccination baseline values.

**Fig 7:**
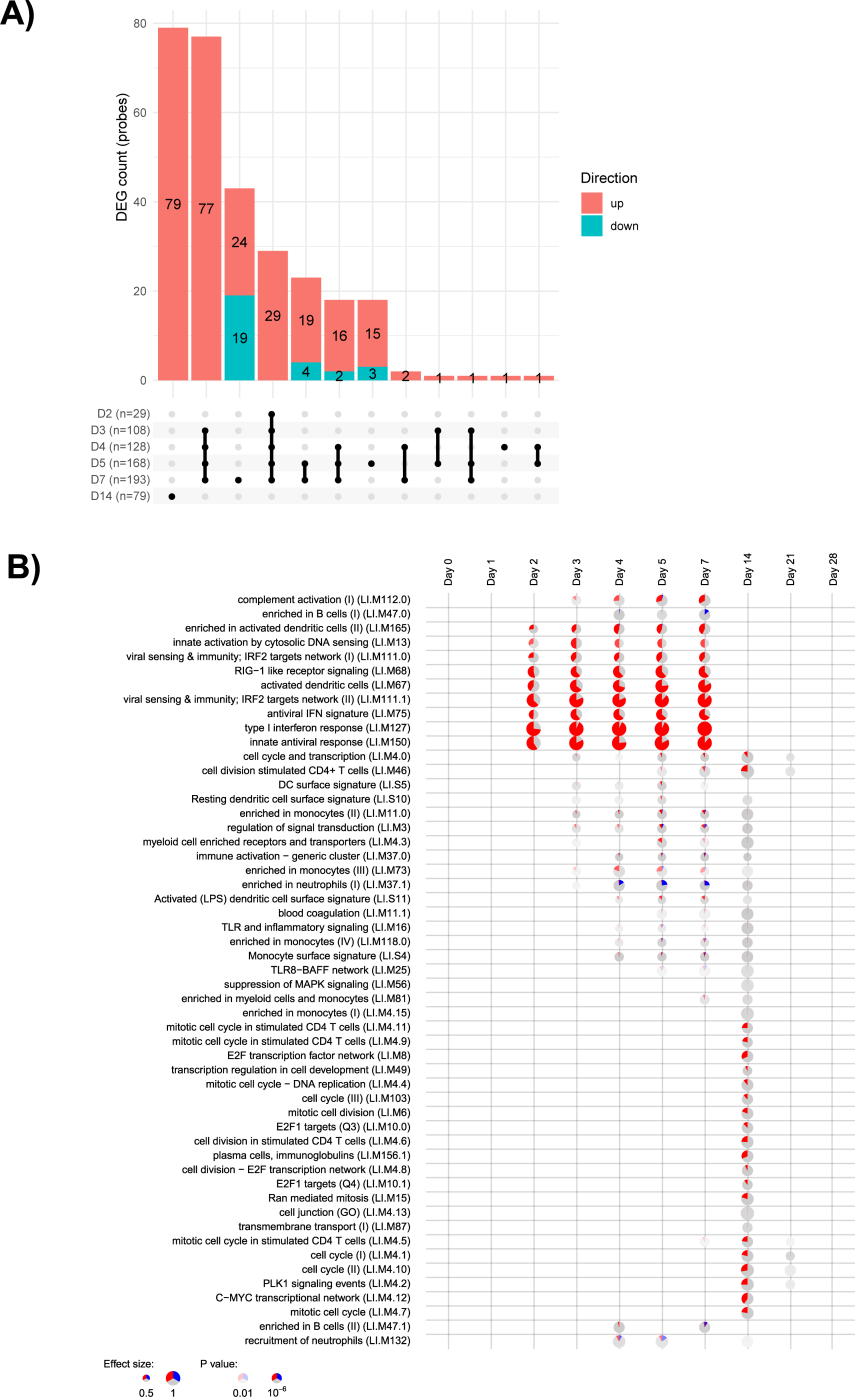
Transcriptomic response to vaccination with YF-17D in humans. (**A**) Upset plots illustrate the number of differentially expressed probes for each time point versus the baseline, and the number of genes in common for each comparison. Genes are considered in common if they have the same fold-change direction. In the bar plot, red represents up-regulated and blue down-regulated genes. (**B**) Gene set enrichment analysis at each time point. The modules (in rows) are described by the titles followed by the original IDs. Each column corresponds to a different time point after vaccination with YF-17D. The size of each pie shows how strong the enrichment is and stronger colors indicate lower *P*-values. Red represents up-regulated and blue down-regulated genes.

**Fig 8:**
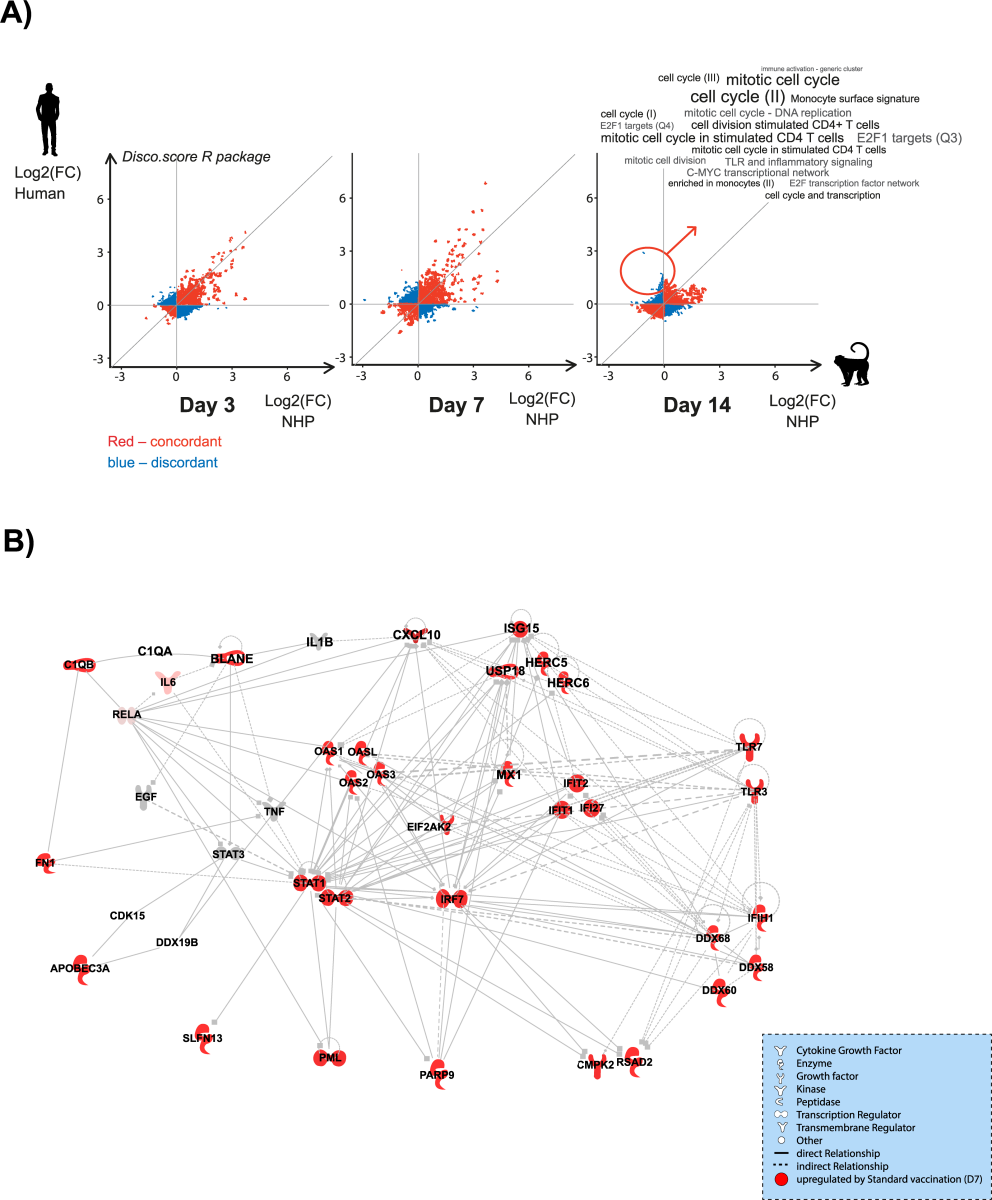
Transcriptomic response to vaccination with YF-17D in humans compared to cynomolgus macaques. (**A**) Distribution of disco.score in the assessment of similarity and dissimilarity of gene expression changes in humans and cynomolgus macaques at different time points after vaccination. Log2-fold changes of gene expression after vaccination in cynomolgus macaques are plotted against log2-fold changes of gene expression in humans. The intensity of the color represents the disco.score. The color illustrates the high degree of similarity (strong red) or dissimilarity (strong blue) in gene expression between the two species. (**B**) IFN network. Ingenuity pathway analysis (IPA) network analysis indicates annotated interactions between genes affected after vaccination with YF-17D (**D7**). Up- (red) regulated genes are indicated.

In human participants, since both placebo and YF-17D vaccine were assessed, the placebo effect measured in the placebo-control group was subtracted from the vaccine group responses (though the analysis in cynomolgus macaques was done without subtraction of the placebo group). There were 251 DEGs (293 probes) across all time points, and the peak occurrence of these DEGs was from D2 to D7 (Fig. 7A; Fig. S6), with similar numbers to those observed in cynomolgus macaques. The upset plots (Fig. 7A) show a common activation process involving the same gene regulatory networks at D2, D3, and D4, and this activity continued at D7, as observed in cynomolgus macaques. A large portion of genes up-regulated at these time points were the same. Gene set enrichment analysis (Fig. 7B) showed that the IFN viral sensing and DC activation modules were enriched and up-regulated from D3 to D7, similar to cynomolgus macaques (Fig. 7B and 8A). None of the gene sets assessed were down-regulated at any time point, which is in contrast with the down-regulation of some genes observed at D1 in cynomolgus macaques.

The first set of enriched BTMs included viral sensing genes such as pattern recognition receptors including Toll-like receptors (TLR) 3 and 7, retinoic acid-inducible gene-I (RIG-I; DDX58), and melanoma differentiation-associated protein 5 (MDA-5; IFIH1). These pattern recognition receptors are known to sense viruses and activate an innate immune response; these sensors also lead to activation of the IFN regulatory transcription factor and subsequent antiviral cascade induction.

The most highly activated pathway following vaccination with YF-17D was the antiviral IFN-related module encompassing 32 closely related IFN and antiviral response genes (Fig. 8B). These genes were mostly down-regulated at D1 in cynomolgus macaques only (in about 50% of cynomolgus macaques) and then up-regulated at D3 and D7 in both cynomolgus macaques and humans. Again, this probably reflects differences in response kinetics between the two species. These modules include genes linked to IFN signal transduction such as IRF7, STAT1, STAT2, and different interferon-stimulated genes (ISG) involved in the direct impairment of viral replication steps: ISG15/HERC5 and Viperin/CMPK2 were among the top 10 DEGs.

In cynomolgus macaques, in addition, the NK cell BTM was down-regulated at D3 after vaccination with YF-17D, which was not observed in humans. This change was very transient, and may simply reflect differences in response kinetics due to the higher vaccine dose-to-weight ratio used in cynomolgus macaques; the same vaccine dose (one human dose) was used in both cynomolgus macaques and humans, with the former on average weighing 10- to 15-fold less. Two other functional categories were up-regulated at D14 in cynomolgus macaques: erythrocyte production, which may be related to blood regeneration in response to the frequent sampling during the study (BTM, heme biosynthesis I and II, and erythrocyte differentiation), and up-regulation of a set of genes that we characterized as neutrophil activation genes, and more specifically of azurophil granule enzymes. As no BTMs specifically included this set of genes, we looked at other gene sets such as modules described by Chaussabel et al. (29) and in the molecular signatures database (MSigDB) gene sets (30). Two gene sets from these additional databases were enriched, neutrophils (DC.M5.15) and “Martinelli immature neutrophil up” (M14418, in the C2 curated gene sets collection, subcollection GCP: chemical and genetic perturbations gene sets), containing genes such LTF, MMP8, MPO, CTSG, and ELANE (Fig. S7) (29, 31). However, it is not clear why neutrophil activation should occur at such a late time point after vaccination.

No up-regulation of the erythrocyte production pathway was observed from D14 to D28 in human participants (Fig. S8). In total, 390 genes corresponding to 427 probes were differentially expressed in the YF-17D group, and 155 genes corresponding to 157 probes in the placebo group. Up-regulation of heme biosynthesis gene sets was observed in both vaccinated and placebo groups, similar to cynomolgus macaques, confirming the probable link with blood regeneration in response to the frequent blood sampling in both species.

Disco.score comparison of human (without placebo correction) and cynomolgus macaque transcriptomics responses to vaccination with YF-17D showed that the gene expression profiles were very concordant at every time point (Fig. 8A). Only two exceptions were observed: (i) at D1 and D14, some genes sets were down-regulated in cynomolgus macaques but not in humans (these corresponded to gene sets such as the antiviral IFN response that was up-regulated at D3 and D7); and (ii) at D14, stimulated CD4 T-cell and cell-cycle modules were up-regulated in humans only. Nevertheless, genes annotated in stimulated CD4 T-cell and cell-cycle gene modules were slightly up-regulated (although not significant) for some cynomolgus macaques at D7 (Fig. S9).

## DISCUSSION

We compared the hematological, biochemical, cytokine, and transcriptome profiles as well as the innate and adaptive immune responses in cynomolgus macaques and adult human participants following the administration of a single dose of the YF-17D vaccine in an attempt to provide new insights into the translatability of the changes observed in the cynomolgus macaque model to human outcomes. To our knowledge, no direct, comparative, parallel head-to-head studies in cynomolgus macaques and humans following the course of YF-17D vaccination have been reported.

As expected, the vaccine had a good safety profile, was well tolerated, and elicited largely similar immune responses in both species. The YF-17D vaccine did not cause any symptoms (including fever) or abnormalities in clinical parameters, and cytokines and hematological/biochemical indices remained relatively unchanged. Our observations are consistent with previous studies in humans (32), and in cynomolgus macaques (33), which reported no significant reaction to the YF-17D vaccine (including other vaccines utilizing the YF-17D backbone) in terms of behavior, weight, food intake and activity, and hematology, serum chemistry, and cytokine parameters remained within normal limits. Very low-level transient vaccine RNAemia (D2 to D6) was detected in 28% of cynomolgus macaques; the RNAemia levels were at least 10,000-fold lower than those observed following infection with the wild-type YFV Asibi strain and lower than the current recommended cut-off standard of 2.7 Log IU/mL (17). RNAemia was generally absent in humans (except for one participant). The difference in detectable RNAemia may be related to the higher vaccine dose-to-weight ratio administered in cynomolgus macaques relative to adult humans. In the literature, vaccine viremia has been reported in 2.2% to 67.5% of recipients of YF-17DD or YF-17D vaccines from D3 to D7 following vaccination (34–36). The variability in reported rates depends on the vaccine used, YF-17D or YF-17DD, the method of viremia detection, that is, viral plaque assay or RT-PCR (34) and the volume of serum used for viremia detection (from 0.14 to 1 mL). However, in some cases, the relevance of the viremia detected from a high volume of serum by qRT-PCR can be debatable as RNAemia below 1 equivalent infectious unit (calculation based on the ratio of 100–1,000 Geq/PFU) has been reported (36). Although RNAemia was not detected in the majority of cynomolgus macaques, or humans, anti-NS1 IgG was detected and is indicative of YF-17D replication. Indeed, activation of the early innate response may depend on virus replication and probably plays a role in the subsequent adaptive immune response and the production of neutralizing antibodies (37).

Our study shows that the progression of the immune response to vaccination with YF-17D in humans appears to follow the same course as described in cynomolgus macaques but with a slightly later onset. For example, YFV-neutralizing antibodies were detected from D14 post-vaccination in adult humans instead of from D7 as in cynomolgus macaques. The higher vaccine dose-to-weight ratio administered to cynomolgus macaques relative to that given to human participants may, in part, also account for the earlier immune response in the former (38). Even though immune response kinetics differed slightly, YFV-neutralizing antibodies reached the same level at the plateau phase at D14 post-vaccination. In addition, circulating blood plasmablasts were detected early post-vaccination in both humans and cynomolgus macaques demonstrating induction of a memory B-cell response. This was expected as the YF vaccine induces long-lived immunity and protective efficacy after a single dose (39).

Similar transcriptomic profiles were observed in both cynomolgus macaques and humans in general. Vaccination with YF-17D up-regulated type I IFN, detection of viral PAMP (TLR, RLR), antiviral innate response, and DC activation pathways at D3 and D7 post-vaccination. In general, most of the up-regulated genes were expected following vaccination with YF-17D and have been previously described in publications of transcriptomics data from human clinical trials (Fig. S10) (22, 23). The minor differences observed at D1 post-vaccination with down-regulation of some genes (19/127) observed only in cynomolgus macaques may be due to delayed baseline sampling before vaccination and/or the higher vaccine dose-to-weight ratio. The neutrophil azurophil granule protein pathway was also up-regulated in cynomolgus macaques only (at D14). The reasons for the up-regulation of neutrophil activation are unclear, although it has been suggested that erythrocytes regulate neutrophil activation (40), and as such, may be related to the decrease of erythrocyte concentration in the bloodstream due to the frequent blood sampling.

Up-regulation of genes coding for different ISGs plays an important role in innate immunity against viral infections, acting directly on viral replication/dissemination control. The IFN-inducible protein, viperin, was shown to inhibit viral replication/propagation through a number of mechanisms including the generation of RNA-dependent RNA polymerase chain terminators (i.e., ddhCTP) and disruption of lipid raft domains thereby preventing viral budding (41, 42). The CMPK2 gene is consistently found next to the viperin gene in vertebrate genomes, and since they are cotranscribed during IFN stimulation, a functional linkage between the two has been proposed (41). Other IFN-inducible proteins have also been shown to have antiviral activity (43, 44). ISG15 activity through ISGylation (45) is implicated in a plethora of antiviral responses such as interference with viral replication through the disruption of host pathways required for oligomerization of viral protein, which can disrupt viral protein function and geometry of the virus, and inhibition of virus egress, as well as in the regulation of host damage/repair responses. Other functions ascribed to ISG include induction of NK cell proliferation, IFNγ and IL-10 production, DC maturation, and acting as a neutrophil chemotactic factor and activator (45).

Although our results in cynomolgus macaques are consistent with those in humans, the response profile appears slightly different from that previously described by Engelmann et al. in rhesus macaques after vaccination with YF-17D (19). Nonetheless, several of the same DEGs were identified in both studies. The time course and number of DEGs may be variable between different NHP species or among the same species; only three rhesus macaques were assessed following vaccination with YF-17D in the study by Engelmann et al., versus 12 cynomolgus macaques in our study, which increases the statistical power to identify a statistically robust list of DEGs.

The whole blood transcriptomic analysis was complemented with the phenotypic analysis of blood cells. We used the CyTOF technology to compare the immune responses elicited in cynomolgus macaques and humans following vaccination with YF-17D. Different marker panels were developed to identify cynomolgus macaque or human cells that comprised 18 common markers, in addition to CD19 for humans or CD20 for cynomolgus macaques, for inter-species comparison. Previous comparison of cynomolgus macaques and human immune cells by mass cytometry in non-vaccinated cohorts had revealed some differences between species such as distinct intensity of expression or co-expression of some markers (18). In particular, monocytes and neutrophils appeared more activated in cynomolgus macaques than in humans as observed in the present study. However, despite the limitations of inter-species immune population comparison, humans and macaques share a common global immune phenotypic profile with respect to YF-17D-induced changes in the frequency of innate and acquired immune system compartments.

We observed that some cynomolgus macaque (29%) and human (11.4%) phenotypic families had a significant increase or reduction in size during the 4 weeks following vaccination with YF-17D. Cynomolgus macaques responded earlier and with greater magnitude than humans. Changes in the frequency of cynomolgus macaque neutrophils, NK cells, monocytes, T cells, and B cells occurred early after vaccination and in most cases, were sustained until the end of the study (4 weeks). In humans, we also observed neutrophil, monocyte, and T-cell frequency changes after vaccination with YF-17D; however, they were of a lower magnitude than in cynomolgus macaques, and immune population expansions or contractions were sustained for no more than 2 weeks. The differences in kinetics and magnitude of immune responses, in terms of cell frequency changes elicited by vaccination with YF-17D, could be explained by the higher antigen dose administered to cynomolgus macaques relative to body weight, as well as by inherent differences between the two species, which influence host-pathogen interactions post-immunization.

The type I IFN-associated viral sensing and DC activation pathways were up-regulated from D3 to D7 in both cynomolgus macaques and humans. In cynomolgus macaques, these coincided with the expansion of HLA-DR^+^CD14^+^ monocytes, characterized by the co-expression of CD32, CD11a, and CD11b that was sustained till D28 post-vaccination. In humans, these could account for the transient expansion from D7 to D14 post-vaccination of two families of DCs, that is, E and F characterized by HLA-DR^+^CD14^–^CD11a, CD11b, and CD123^–^ or ^+^, respectively. The temporal association observed between these two events suggests that the up-regulation of antiviral gene expression occurs in monocytes or DCs, or alternatively that expansion or activation of these cell populations are modulated by the IFN response. In addition, in response to the type I IFN pathway activation, we observed down-regulation of the NK-cell blood transcriptomic module in cynomolgus macaques at D3, which contrasted with the expansion of the NK “family D” observed as early as D1 and remaining elevated (or continued to increase) until D28.

Neutrophil activation genes were up-regulated on D14, coinciding with a significant expansion of the neutrophils (CD66^+^CD11b^+^ “family T”) in humans. However, these neutrophils did not display a “typically” activated phenotype. In contrast, three neutrophil (CD66^+^CD11b^+^) families (F, H, and J) in cynomolgus macaques had expanded early (by D1), remained elevated as late as D28, and displayed an activated phenotype, with high expression of CD32a, CD11a, CD62L, and CD89. It remains to be clarified why neutrophil populations that expand early after vaccination persist for so long. Recent studies have shown that phenotypic changes in neutrophils, induced by modified vaccinia virus Ankara immunization in cynomolgus macaques, can persist for at least 56 days post-immunization (46).

The immune responses elicited by vaccination with YF-17D were concomitantly observed by CyTOF and transcriptomics analysis, but this does not correspond to a statistical correlation between the analyses. In addition, since we focused on whole blood analysis we likely missed features associated with some cell populations or responses associated with migration/attraction to the site of immunization and/or to other tissue sites.

There are some limitations to our study. We did not assess transcriptional dynamics in cynomolgus macaque placebo controls; ethical considerations did not allow for the use of a placebo. The samples collected before vaccination with YF-17D served as individual baseline for each cynomolgus macaque and it is possible that some of the observations (including the up-regulation of erythrocyte production) could be, in part, due to the response to the study procedures. In addition, also for ethical considerations, the baseline sampling in cynomolgus macaques was performed 3 to 4 weeks before the vaccination day; baseline sampling closer to the vaccination day would improve the observed baseline heterogeneity notably in transcriptomics analysis. We did not harmonize some of the methods used to assess samples from cynomolgus macaques and human participants and some analytical read-outs were only available on small subsets. For example, for practical reasons, different technologies were used for transcriptomic analyses, RNASeq for cynomolgus macaques, and DNA array in human participants. Strengths of our study include the parallel head-to-head study design in cynomolgus macaques and humans to allow for a more direct and meaningful comparison of the data between the two species. The sample sizes used were sufficiently large to allow for meaningful statistical comparisons.

In conclusion, we demonstrated that the YF-17D vaccine has a good safety profile and largely elicited similar gene expression profiles and immune responses in both cynomolgus macaques and human participants. This study, therefore, confirms that cynomolgus macaques are a valid model for replicating YF vaccine responses in humans.

## MATERIALS AND METHODS

### Study designs

**Fig 9:**
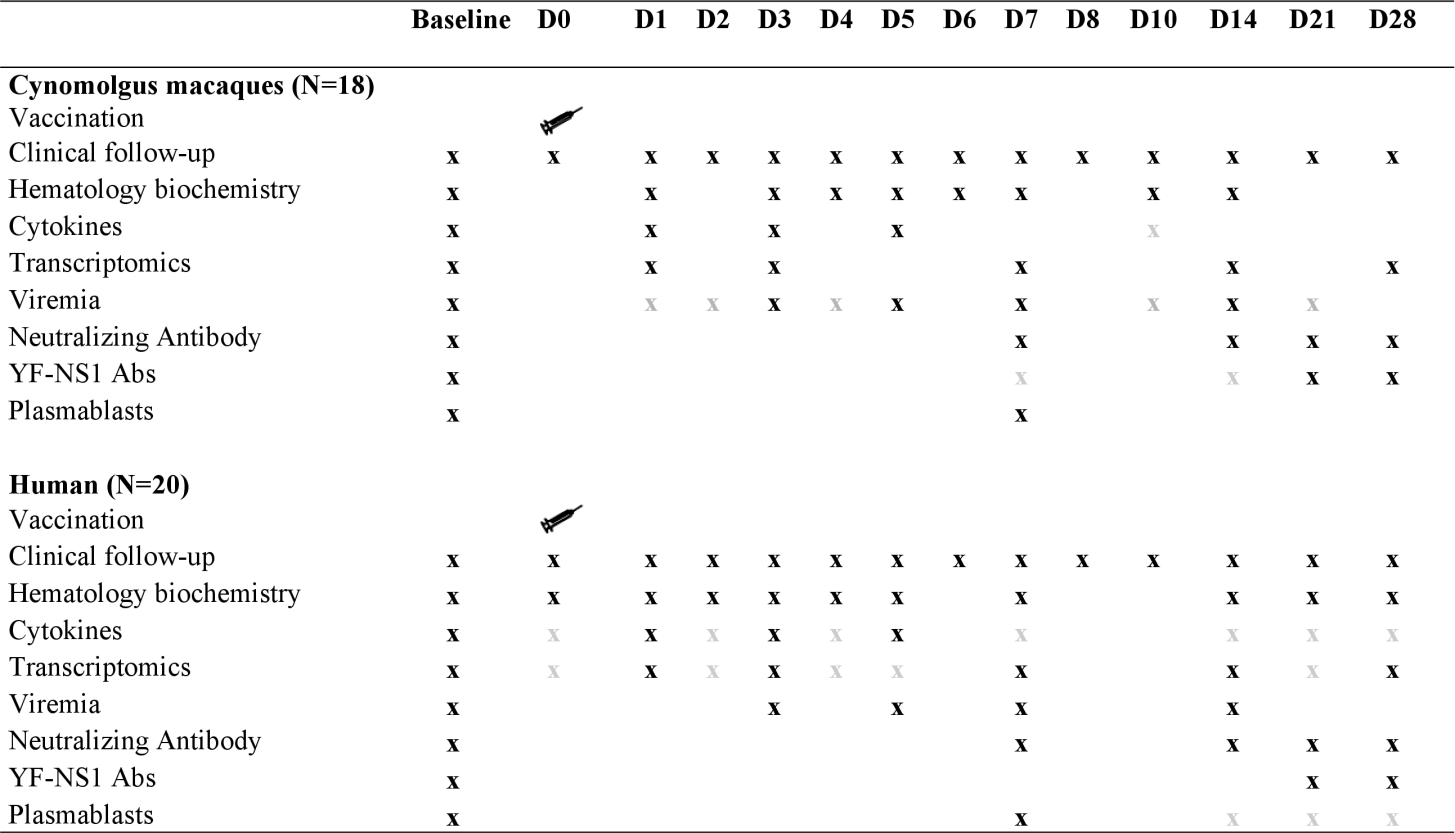
Study design. Black cross: common to the two studies, can be used for human/cynomolgus macaques comparison. Gray cross: not common, not used for comparison.

These studies were undertaken within the BIOVACSAFE framework (Biomarkers for enhanced vaccines immunosafety; www.biovacsafe.eu) and have been described in part elsewhere (18, 26). Parallel studies in human participants and cynomolgus macaques (taking into account ethical guidelines) with similar sampling times and analyses undertaken before and after vaccination with YF-17D (Fig. 9) were performed to allow for direct translational assessment that can better link findings between species and identify intrinsic differences. Such a methodological approach would assist in the interpretation of data collected and help identify markers in cynomolgus macaques that could be highly predictive of clinical outcomes.

### Vaccine and placebo

The live, attenuated YF-17D-204 vaccine (monodose Stamaril, Sanofi) was presented as powder and diluent for reconstitution immediately before use in 0.5 mL dose volumes containing not less than 1,000 IU for both human participants and cynomolgus macaques. The diluent was 0.4% sodium chloride solution. The placebo was 0.9% sodium chloride solution (0.5 mL). Allocation of vaccine or placebo in human participants was randomized (20 and 8 received vaccine and placebo, respectively) and administered subcutaneously in the deltoid region of the left upper arm.

### Recruitment and clinical safety monitoring

The vaccine recipient participants were healthy adults (13 males and 7 females), aged 21–42 years (mean 29 years), with a mean BMI of 23.7 kg/m^2^, recruited in the UK in 2013. Participants were deemed eligible based on negative YF vaccination history and serology, and no significant abnormality at screening on medical history, physical examination, vital signs or hematology, biochemistry, and urinalysis profiles. All were admitted as residents at the Surrey Clinical Research Center on the day before vaccination and for 5 days post-vaccination and re-attended on D7, D14, D21, and D28 post-vaccination. Whole blood and serum were obtained the day before and immediately before vaccination, daily for 5 days post-vaccination, and on all subsequent scheduled visits to the research center; timelines for the acquisition of samples are presented in Fig. 9. The placebo recipient participants (*n* = 8; matched for age, race, and gender) were followed-up in the same manner.

Reactogenicity, local, and systemic unsolicited adverse events (AEs, classified using the MedDRA-preferred terms) were recorded throughout the study. Injection site complications from indwelling cannulas such as phlebitis were checked daily and recorded, but excluded from reactogenicity calculations. Participants’ vital signs (pulse, blood pressure, temperature) were checked on a daily basis during the inpatient stay and at each outpatient visit, and they were also queried for the occurrence of any AE, both solicited and unsolicited, but there were no symptoms of interest were specifically solicited.

### Cynomolgus macaque cohorts

Cohort 1 comprised 12 male cynomolgus macaques (*Macaca fascicularis*; Noveprim, Mauritius) aged 2.7–5.4 years (mean 3.5 years) and weighed 3.9 to 7.1 kg (mean 4.5 kg) at the start of the study. The cynomolgus macaques were housed at the IDMIT (CEA, Fontenay-aux-Roses, France) in collective cages based on social affinity by the supplier (scoring grid) and allowed to acclimatize for 5 weeks before study procedures. The macaques were monitored and fed 1–2-times daily with commercial monkey chow and fruits, and water *ad libitum*. Environmental enrichment provided included toys, novel foodstuffs, and music. After vaccination with YF-17D, the cynomolgus macaques were housed in individual adjoining cages to allow social interactions, and under controlled humidity, temperature, and light (12-hour light/12-hour dark cycles).

Cohort 2 comprised of six male cynomolgus macaques (*Macaca fascicularis*; Noveprim, Mauritius) aged 2–3 years and weighed 2.8–3.0 kg at the start of each study. These cynomolgus macaques were housed in facilities at Sanofi (Marcy L’Etoile, France) with appropriate enrichment as described for cohort 1. The cynomolgus macaques were housed in collective cages based on social affinity and allowed to acclimatize for 1 month before study procedures.

For both cynomolgus macaque cohorts, experimental procedures (animal handling, immunizations, inoculations, blood samplings) were conducted after animal sedation with ketamine chlorydrate (Rhône-Mérieux, Lyon, France, 10 mg/kg, intramuscular route) or zolazepam-tiletamine (Zoletil 100, Virbac, Carros, France, 0.05 mL/kg, intramuscular route). Blood samples were obtained 21 (cohort 1) or 28 (cohort 2) days before vaccination and then daily after vaccination for the first eight time points (D1 to D8), and then at D10, D14, D21, and D28. An overview of the timelines for the acquisition of samples from cynomolgus macaques is presented in Fig. 9.

At the end of the studies, the cynomolgus macaques were sedated with ketamine chlorhydrate or zolazepam-tiletamine and then euthanized by intravenous injection of 180 mg/kg sodium pentobarbital.

### Serum cytokines

Serum cytokine concentrations were assessed using Luminex multiplex assay kits according to the manufacturer’s instructions. For human participants, a 16-cytokine custom kit purchased from R&D multiplex kit (Catalog Number LXSAHM: TNF-alpha, IL-8, MCP1, IFN-gamma, MIP1-alpha, IL-1alpha, GM-CSF, IP10, TNF-R1, IL6, IL-10, VEGF, PTX3, IL-2, IL-5, IL-2RA) and three cytokine custom kit purchased from Millipore (IFN-alpha2; IL-12-p40; IL-1RA) were used. For cynomolgus macaques, the Plex NHP Cytokine/Chemokine/GF 37 Plex Procarta (ref: EPX-370–400-45-901) multiplex kit purchased from Thermo Fisher was used. The NHP kit measures 37 protein targets: BDNF; BLC (CXCL13); NGF beta; Eotaxin (CCL11); FGF-2; G-CSF (CSF-3); GM-CSF; IFN-alpha; IFN-gamma; IL-1 beta; IL-10; IL-12p70; IL-13; IL-15; IL-17A (CTLA-8); IL-18; IL-1RA; IL-2; IL-23; IL-4; IL-5; IL-6; IL-7; IL-8 (CXCL8); IP-10 (CXCL10); I-TAC (CXCL11); MCP-1 (CCL2); MIG (CXCL9); MIP-1 alpha (CCL3); MIP-1 beta (CCL4); PDGF-BB; sCD40L; SCF; SDF-1 alpha (CXCL12a); TNF-alpha; VEGF-A; and VEGF-D. The data were acquired with the Bio-Plex−200 system and Xmap platform (Luminex Corporation), equipped with the Bio-Plex Manager software, and expressed as acquired mean fluorescence intensity after background subtraction. The lower and upper quantification limits for the assays were determined based on the standard curve for each cytokine and each run.

### Hematology and blood biochemistry

In both human participants and cynomolgus macaques, blood hematology was assessed on the day of collection, with sera collected for biochemistry analysis and stored at ≤−70°C until analysis.

Cynomolgus macaque hematology analyses (red blood cells, hemoglobin, hematocrit, mean cellular volume, mean corpuscular hemoglobin, mean corpuscular hemoglobin concentration, platelets and white blood cells including lymphocytes, monocytes, neutrophils, eosinophils, and basophils) were determined using EDTA-treated blood samples with the HMX A/L (Beckman Coulter) and DxH 800 (Beckman Coulter) hematology analyzer. Serum biochemistry (alkaline phosphatase (ALP), alanine aminotransferase (ALT), gamma glutamyltransferase (GGT), total bilirubin (TBIL), albumin (ALB) blood urea nitrogen (BUN), creatinine, and C-reactive protein (CRP) analyses were realized by Vebio (Arcueil, France) for cynomolgus macaques. For human samples, hematology and biochemistry assessments were undertaken by the accredited clinical pathology department at the local hospital.

### YF-17D RNAemia

YF-17D RNA was quantified in plasma samples obtained at baseline and on D3, D5, and D7 in the clinical study and on D2, D3, D4, D5, D6, D7, and D10 in the cynomolgus macaque study. Total RNA from plasma samples was extracted using a Nucleospin 96 virus kit (Macherey Nagel cat # 740691) on the TECAN Evoware platform as per the manufacturer’s instruction, and YF-17D RNA copy numbers determined by qRT-PCR based on the detection of the NS5 gene fragment, as described previously (47). The limit of detection (LOD) and limit of quantification (LOQ) of these assays were 3.0 Log genomic equivalents (GEQ)/mL (i.e., 1 to 10 plaque-forming units (PFU)/mL) and 3.3 Log GEQ/mL, respectively. Samples where no virus was detected were assigned a value of 2.7 Log GEQ/mL (i.e., half the LOD) for geometric mean concentration (GMC) calculations.

### YFV plaque reduction neutralization test

Neutralizing antibody levels against the YF-17D strain in clinical serum samples were determined with the 50% plaque reduction neutralization test (PRNT_50_) before vaccination and on D7, D14, D21, and D28 post-vaccination. Twofold serial dilutions of heat-inactivated serum starting at 1:5 dilution were mixed with a challenge dose of YF-17D virus (400 PFU/mL) and inoculated in duplicate into 24-well plates of confluent Vero cells. After inoculation, the confluent Vero cells were overlaid with a semi-solid medium containing methyl cellulose and incubated for a few days.

The cynomolgus macaque serum samples obtained at the same time points as for the human participants were analyzed in a similar manner using the miniaturized 50% plaque reduction neutralization test (µPRNT_50_). Twofold serial dilutions of heat-inactivated serum starting at 1:5 dilution were mixed with a challenge dose of YF-17D virus (4,000 µPFU/mL) and inoculated into 96-well plates of confluent Vero cells. The cell monolayers were incubated for 45 hours.

The plaques in both neutralization assays were visualized by immunostaining with flavivirus-specific monoclonal antibodies 4G2. The neutralizing antibody titer was calculated using the least square method and corresponds to the reciprocal of the dilution demonstrating a neutralization of 50% of the plaques compared with the virus-alone control wells. The LLOQ was 10 in both neutralization assays, corresponding to the starting dilution of the µPRNT method.

### FluoroSpot/cytometry

Peripheral blood mononuclear cells (PBMCs) were isolated from blood collected in heparin CPT tubes (BD Vacutainer, BD) according to the manufacturer’s instructions. Those isolated from human participants were cryopreserved until analysis, and those from cynomolgus macaques were analyzed on the same day. The PBMCs were resuspended in RPMI 1640 medium with glutamax-1 (Gibco, Paisley, UK) supplemented with 10% heat-inactivated fetal bovine serum (FBS; Laboratoires Eurobio) before analysis.

#### *Ex vivo* B-cell FluoroSpot (cynomolgus macaques)

The numbers of IgM and IgG-producing B cells in freshly isolated PBMCs were assessed with the Monkey FluoroSpot IgG/IgM kit (Mabtech, ref FS-05R17G-2) according to the manufacturer’s instructions. In brief, the membranes of 96-well FluoroSpot microplates equipped with a low-fluorescent polyvinylidene difluoride (PVDF) membrane (Merck Millipore) were pre-wetted for 45 seconds with 50 µL of 35% ethanol. Each well was washed four times with 200 µL of sterile water and once with sterile 1X phosphate-buffered saline (PBS; Laboratoires Eurobio, Les Ulis, France). The plates were then coated with 100 µL of YF-17D infected Vero cell lysate at 9.7 GEq/mL or a mix of monoclonal antibodies specific for monkey IgG and IgM at a dilution of 15 µg/mL, and incubated overnight at +4°C. After incubation, the plates were washed with PBS and blocked for 2 h at +37°C with Roswell Park Memorial Institute medium (RPMI) 1640 supplemented with 10% FBS. The plates were washed again with PBS and PBMCs were added to the wells coated with YF-17D-infected Vero cell lysate (2 × 10^5^ or 4 × 10^5^ cells/well) as well as those coated with anti-IgG and anti-IgM antibodies (0.5 × 10^5^ and 2 × 10^5^ cells/well) and incubated for 16 hours at +37°C in 5% CO_2_. The plates were washed six times with PBS (0.5% FBS), after which 100 µL/well of the anti-monkey IgM-FITC and IgG-CY3 antibodies were added at a dilution of 1:500 in PBS (0.5% FBS) and incubated in the dark for 2 hours at +37°C. The plates were washed six times with PBS (0.5% FBS) and stored at room temperature in the dark until reading. The spots were counted using an Automated Elispot Reader System ELR08IFL (Autoimmun Diagnostika GmbH, Strassberg, Germany). Results were expressed as numbers of antibody-secreting cells per 10^6^ cells.

#### Plasmablasts phenotyping by flow cytometry in humans

Cryopreserved PBMCs were thawed and left overnight at 37°C before use. The PBMCs were stained with CD19-BV421 clone HIB19 (562440), CD38-APC clone HB7 (345807), CD3-BV650 clone UCHT1 (563862), CD20-PerCP clone L27 (345794), and anti-CD27-PE clone L128 (340425) (all antibodies were purchased from BD Biosciences). The cells were incubated with the antibodies for 20–30 min at +5°C ± 3°C. Flow cytometry analyses were performed with FlowJo software. Plasmablasts were gated as CD19^+^CD3^−^CD20^−/low^CD27^high^CD38^high^ on an extended lymphocyte gate to include blasting cells.

### CyTOF

Blood collection, fixation, and storage were undertaken as previously described (18). In brief, whole blood was collected in lithium-heparin vacutainer tubes (BD Biosciences, San Jose, CA) and cells were fixed within 3 hours of blood withdrawal. Fixation mixture (DPBS/formaldehyde 5%, glycerol 18.75%) used to store cells was prepared extemporaneously as previously described (48). Briefly, two parts of double concentrated Dulbecco’s phosphate buffer (DPBS) prepared from a solution of DPBS modified 10X without CaCl_2_ and MgCl_2_, pH 7.4 (Gibco by Life Technologies, Villebon-Sur-Yvette, France), one part 20% formaldehyde prepared from 36% formaldehyde (VWR BDH Prolabo, Fontenay-sous-Bois, France), and one part 75% glycerol (Sigma Aldrich, Lyon, France) were mixed. The resulting solution was stored at +4°C and used within three days. Fixation solution (DPBS/PFA 1.6%) used for the staining was prepared by diluting 16% paraformaldehyde (PFA; Electron Microscopy Sciences, Hatfield, USA) in DPBS modified 10X and Milli-Q water. Staining Buffer (DPBS/BSA 0.5%) was prepared by solubilization of BSA (Sigma Aldrich, Lyon, France) in DPBS modified 1X (Gibco by Life Technologies) at a final concentration of 0.5%.

Blood samples were processed according to a freezing procedure allowing the recovery of all blood leukocytes, especially polymorphonuclear cells. We adapted a previously described cell preparation procedure consisting of fixation, red cell lysis, and freezing (48). Whole blood (1 mL) was mixed with fixation mixture (10 mL) and placed on ice for 10 min. After centrifugation, red cells were lysed by adding 10 mL of Milli-Q water at room temperature (RT) for 20 min. After two washes with DPBS modified 1X, the cells were counted and stored at –80°C in a fixation mixture at a final concentration of 15 × 10^6^ fixed leukocytes/mL and distributed in aliquots containing 3 × 10^6^ cells.

#### Antibody-element conjugation

Antibody-element conjugation was performed as previously described (49). Pure (carrier-protein–free) monoclonal or polyclonal antibodies from various manufacturers were coupled to elements using MAXPAR lanthanide labeling kits (Fluidigm, San Francisco, CA), as indicated in the manufacturer’s preload method for 400 mg of antibody. The antibody-element combinations are shown in Fig. S11A for analysis of human samples and Fig. S11B for cynomolgus macaque samples. Antibody-element conjugates were adjusted to 1 mg/mL in antibody stabilizer buffer (Candor Bioscience, Wangen, Germany), supplemented with sodium azide (Santa Cruz Biotechnology) to a final concentration of 0.01%, and stored at +4°C in sterile conditions throughout the study. Antibody-element combinations were titrated and tested in CyTOF using cells from healthy humans to obtain optimal staining concentrations.

#### Staining and CyTOF acquisition

Staining and CyTOF acquisition were undertaken as previously described (18). In brief, cryopreserved fixed cells (3 × 10^6^ cells) were thawed at 37°C, washed twice with staining buffer, and placed on ice for 30 min with the metal-labeled surface antibodies listed in supplementary materials (Fig. S11A and B). Because poor staining was obtained for B cells from cynomolgus macaques with anti-human CD19 screened monoclonal antibodies, characterization of B-cell phenotypes was performed using the CD19^+^HLADR^+^ and CD20^+^HLADR^+^ combinations for humans and cynomolgus macaques, respectively. After two washes with modified DPBS 1X, the cells were fixed with fixation solution at room temperature for 20 min and permeabilized with 1X Perm/wash buffer (BD Biosciences) at room temperature for 10 min. Intracellular antibodies and iridium nucleic acid intercalator were placed on ice for 30 min. After two washes with DPBS modified 1X, cells were fixed again with fixation solution at room temperature for 20 min, centrifuged, and stored overnight with 0.1 µM iridium nucleic acid intercalator in fixation solution. The following day, the cells were washed with Milli-Q water, resuspended in 1 mL of Milli-Q water, and filtered with a 35 µm nylon mesh cell strainer (BD Biosciences), prior to the addition of EQTM Four-Element Calibration Beads (Fluidigm, San Francisco, USA) according to the manufacturer’s instructions. Each sample was split into two and distributed in a 96-well microplate (Sigma Aldrich, St Louis, MO). The acquisition was done using the autosampler (Fluidigm) device to automatically deliver samples into the CyTOF instrument (Helios, Fluidigm) following standard procedures, as previously described (50).

#### CyTOF data processing

Cytometry data were normalized with Finck’s MATLAB normalizer using the signal from the 4-Element EQ beads (Fluidigm) as recommended by the software developers (51). Replicates were concatenated with the FCS file concatenation tool (Cytobank, Mountain View, CA). Four-Element Calibration Beads were excluded by manual gating on the Ce140 channel. Singlets were selected on an Ir191 (DNA intercalator)/Cell length bivariate dot plot. For human samples, eosinophils, with a strong background in all channels, were excluded on a CD66^+^/CD3^+^ bivariate plot (Fig. S11C).

Two SPADE were independently performed for humans and cynomolgus macaques, using 17 clustering markers (Figure S11A and S11B). SPADE is a clustering method developed to analyze and visualize high-dimensional cytometry data. First, SPADE performs density-dependent down-sampling to equalize the density of the cell cloud and achieve equal representation of rare and abundant cell types. Second, SPADE performs agglomerative clustering to partition the down-sampled cloud into clusters of cells with similar phenotypes. The user-defined steps of the analysis pipeline involve (i) exclusion of doublets, background events, and CD3^+^CD66^+^ aberrant cells (Fig. S11C) (ii); selection of SPADE clustering markers [CD3, CD11b, CD11c, CD14, CD16, CD19, CD28, CD32, CD64, CD66, CD86, CD123, CD184 (CXCR4), CD195 (CCR5), HLADR, Granzyme B, and Perforin] (Fig. S11); and (iii) selection of the desired number of clusters (300 clusters) and the level of density-based down-sampling (25%).

We used the SPADEVizR R-package for the phenotypic analysis of the SPADE clusters (52). Marker intensities were uniformly divided into five categories based on the 2nd and 98th percentile range expression. Hierarchical clustering was performed using the Euclidian metric and the complete linkage method. For better visualization, all clusters were distributed into four groups (granulocytes, monocytes and DCs, T cells and NK cells, and B cells). Independent correlation analysis between human and cynomolgus macaque clusters was performed for each group and was generated using the Manhattan distances based on the relative expression of all 17 common markers (CD66, HLADR, CD3, CD64, CD32, CD16, CD23, CD123, Granzyme B, Perforine, CD11a, CD11b, CD14, CD86, CD28, CCR5, and CXCR4) and CD19 for humans or CD20 for cynomolgus macaques (Fig. 5A).

### Whole blood RNA sequencing

Whole blood from cynomolgus macaques was collected in PAXgene tubes (BD762165—Ozyme) on D –21 cohort 1 or D –28 cohort 2 (baselines), and on D1, D3, D7, D14, and D28, and stored according to the manufacturer’s protocol. RNA was extracted with the PAXgene 96 Blood RNA kit (Qiagen-762331) according to the manufacturer’s instructions on the Tecan Evo150 workstation. The RNA quantity, purity, and quality were checked on the Labchip (Caliper) and the Nanodrop Spectrophotometer (Thermo Scientific). Extracted RNA samples with an A260/A280 ratio ≈2, a RIN > 8, and a minimal concentration of 70 ng/µL of RNA were processed using the GLOBINclear-Human kit (ThermoFisher—AM1980) for the depletion of the alpha and beta globin mRNA according to the manufacturer’s instructions. The quantity, purity, and quality of the RNA depleted of globin mRNA were reassessed as already described before library preparation for sequencing. Sequencing libraries were prepared with the TruSeq mRNA stranded kit (Illumina—20162122/20146602) using 300 ng of RNA according to the manufacturer’s instructions. The quality, length distribution, and quantity of the resulting libraries were analyzed with the Qubit 3.0 Fluorometer (Life Technologies) and the Labchip (Caliper). Libraries were pooled at equimolar amounts and sequenced by single reads of 75 bp on high-output flowcell (Illumina—FC-404-2005) using the Nextseq500 sequencer (Illumina) in a batch of 10 samples per flowcell with the specification to achieve 30 million reads per sample. On run completion, libraries were demultiplexed, adapters were trimmed, and FASTQ files were generated.

### RNA-sequencing data analysis

A read quality check was performed on raw data in FASTQ format using FastQC (v0.11.2). Reads were imported and then processed in OmicSoft Array Studio software (v9.0.6.38-v9.0.8.28). The raw data QC wizard of Array Studio was executed on the reads to assess the read quality. The reads were aligned with OmicsSoft Aligner [OSA4 (53);] in Array Studio against the Cyno.WashU2013 reference genome using WashUGene20140620 gene annotations, including a first step of read trim per quality score (minimum of 2). Samples (CCB102J-21, CCA100J1, CCB102J1, CCA100J14, CCA100J-21, CA100J7, CCA100J3, CCB102J3, and CCA100J28) that did not meet quality criteria based on the minimal number of reads sequenced underwent a second run, and files were merged after read alignment, that is, at the BAM file level.

Naïve read count quantification was performed with the Array Studio software using WashUGene20140620 gene annotations (In Array Studio, “Naïve” means that no EM algorithm was used to handle ambiguous reads). Multiple mapping reads and first-read-forward-strand reads were discarded from the analysis (i.e., “Exclude multi-reads” option selected, “Count First-Read-Forward-Strand reads” option unselected). Read counts were then subsequently analyzed using R scripts (R version 3.3.3). Genes exhibiting low counts across all samples were removed using the filterByExpr.R function of the edgeR package (https://rdrr.io/bioc/edgeR/src/R/filterByExpr.R, introduced in edgeR v3.21.2). All samples were normalized with TMM using the calcNormFactors function of the edgeR package (v3.16.5 (54, 55)) and transformed with voom (Limma R package v3.30.13).

For the linear modeling of differential gene expression, a model was built with Limma using the sampling time point as a factor (relative to the vaccination day). Contrasts were set up to compare each time point post-vaccination with baseline (D –21) (56, 57)). The Limma duplicateCorrelation function was used to estimate the correlation between measurements made on the same subject. The option robust = TRUE was selected when running eBayes. The TopTable function was used to select differentially expressed genes on each contrast separately with a statistical cutoff of the Benjamini-Hochberg (BH) adjusted p-value less than 0.05 and an absolute fold change over 2.

The Genbank database (58) gene group file was used to identify NHP to human orthologs (1st June 2017; ftp://ftp.ncbi.nlm.nih.gov/gene/DATA/gene_group.gz) for each gene available in the WashUGene20140620 gene annotations, using Entrez Gene identifiers. Corresponding human gene symbols were then retrieved using the Genbank Homo_sapiens.gene_info file (June 2nd 2017; ftp://ftp.ncbi.nlm.nih.gov/gene/DATA/GENE_INFO/Mammalia/Homo_sapiens.gene_info.gz).

### TMOD enrichment

The tmod R package (v0.31 (59)) was used to detect enrichment directly on the Limma object (i.e., inference test result from the Limma R package) on the full genes list (ordered using a default “minimal significant difference” ranking) coming from each contrast. Enrichment analysis was performed using BTMs established by Chaussabel et al. (29), Li et al. (28), and the MSIgDB gene sets C2 (v6.2 (30)). As the tmod annotations were not updated recently, tmod gene symbols were matched to the latest gene symbols using Genbank Homo_sapiens.gene_info file information and tmod Entrez Gene identifiers. Tmod panel plots were displayed for all the BTMs from Li et al. (28) with an adjusted *P*-value under 0.0001. The results for each comparison test were shown side by side on a tmod panel plot for information purposes.

#### Gene interaction network analysis

Outcomes from Limma-voom analysis were first uploaded into the Ingenuity Pathway Analysis (IPA) software program (QIAGEN Inc., https://www.qiagenbioinformatics.com/products/ingenuitypathway-analysis) (60). Differentially expressed genes (with a fold-change greater than X and an adjusted *P*-value less than X) were investigated with IPA, to build a gene interaction network based on known interactions from the literature for specific biological functions.

#### Human microarray data generation

Human microarray data generation was undertaken as previously described (26). In brief, peripheral blood was drawn into PAXgene tubes (PreAnalytiX) and RNA was extracted on the automated QIAcube system (Qiagen) using the PAXgene Blood RNA kit (Qiagen) according to the manufacturer’s instructions. Quality control and quantification of isolated RNA were analyzed with an Agilent 2100 Bioanalyzer (Agilent Technologies) and a NanoDrop 1000 UV-Vis spectrophotometer (Thermo Fisher Scientific). Microarray experiments were performed as single-color hybridization, and RNA was labeled with the Low Input Quick-Amp Labeling Kit (Agilent Technologies). mRNA was reverse transcribed and amplified using an oligo-dT-T7 promoter primer and labeled with cyanine 3-CTP by T7 *in vitro* transcription. After precipitation, purification, and quantification, 0.75 µg labeled cRNA was fragmented and hybridized to custom whole genome human 8  ×  60 K multipack microarrays (Agilent-048908) according to the supplier’s protocol (Agilent Technologies). Scanning of microarrays was performed with 3 µm resolution and 20-bit image depth, using a G2565CA high-resolution laser microarray scanner (Agilent Technologies). Microarray image data were processed with the Image Analysis/Feature Extraction software G2567AA v. A.11.5.1.1 (Agilent Technologies), using default settings and the GE1_1105_Oct12 extraction protocol.

#### Microarray normalization and quality control

Blinded primary readouts of the microarrays were read, background corrected, normalized, and controlled for quality using the R package limma41 (version 3.30) as previously described (26). For background correction, the gProcessedSignal from the primary readouts was used. Between-array normalization was done using the quantile method in limma. Quality control relied on density plots, testing for outliers, visualization with principal component analysis, and visual inspection of individual array images. The normalized data were locked and unblinded for further analysis. All primary readouts and the background corrected and normalized data are available in the Gene Expression Omnibus (GEO) database under the BioProject identifier PRJNA515032.

#### Human microarray data analysis

Agilent microarrays were processed using R scripts (R version 3.3.3) and the Limma R package (v3.30.13). Between-array normalization was applied with the quantile method from Limma. Three arrays were removed from the analyses as their quality checks were not sufficient. Duplicate probes were averaged, and lincRNAs were removed from the analysis.

Two types of contrasts were applied, in each case comparing to D –1, one subtracting the placebo effect from the YF-17D effect (e.g., (YF-17D [D7] – YF-17D [D –1]) – (Placebo [D7] – Placebo [D –1]), the other considering each group separately to check for placebo-specific effects and compared with cynomolgus macaques. Similar to cynomolgus macaques, the duplicate Correlation function was used to estimate the correlation between measurements made on the same participant.

#### Concordance and discordance between humans and cynomolgus macaques using the disco.score

Disco R package (61) was used for an in-depth investigation of the concordance and discordance of module enrichment across cynomolgus macaques and humans. For each homologous gene pair, the degree of change in gene expression (log-fold change), statistical significance of the differential expression (*P*-values), and direction of differential expression were used to calculate the disco.score.


disco.score: =|log2FC1|⋅|log2FC2|⋅(|log10(p1)|+|log10(p2)|)⋅sign(logFC1)⋅sign(logFC2)

- log2FC1 log2 fold change of the expression change of the gene from cynomolgus macaques
- log2FC2 log2 fold change of the expression change of the gene from humans
- p1 differential regulation adjusted *P*-value for the gene from cynomolgus macaques
- p2 differential regulation adjusted *P*-value for the gene from humans

The disco.score increases proportionally to both log2FC (FC 1 or FC 2) increase (or decreases analogously), increases with the decrease in summed *P*-values of gene pairs, and has a negative sign if the expression change has the opposite direction. Once the disco.score is calculated, a BTM module enrichment analysis is run using a gene ranked by disco.score similarly to using the tmod R package (59). Concordant modules are obtained by running gene set enrichment analysis on genes ranked from the highest to the lowest disco.score. Discordant modules are obtained on the gene list ranked in the opposite direction. Tag clouds representations are used to show how BTMs are enriched. In these plots, the size of the BTM’s name is linked to the AUC (effect size), and the color to the *P*-value (light gray to black scale).

#### Human public microarray data analysis

The data from Querec et al. (23) (GSE13486), Hou et al. (24) (GSE82152) as well as from Gaucher et al. (22) (GSE13699) were retrieved from Gene expression omnibus (GEO) and uploaded to ArrayStudio (Omicsoft, Cary, NC, United States) for gene expression analysis. The microarray probes were annotated with GPL7567, GPL21975, and GPL6883. Microarray data were normalized using the Robust Multi-array Average (RMA) method (62) implemented in Array Studio. The Limma approach (56) implemented in Array Studio was used to identify differentially expressed genes (FDR < 0.05; absolute fold-change greater than 2).

### Statistical analyses

The analyses were undertaken on SAS v9.4. All read-outs were log10 (or Log) transformed prior to the statistical analyses. For each read-out, the response elicited by vaccination with YF-17D was compared to the baseline of the read-out (time before vaccination) to determine whether there was a vaccination effect. Descriptive analysis was performed for cytokines, blood hematology, and biochemistry parameters. Analysis of variance with one factor (Time) under the mixed model was performed on longitudinal data for CRP and around 10 cytokine read-outs. For YFV-neutralizing antibodies, a longitudinal mixed model with two factors (Species and Time) as well as the interaction between the two factors was used. For these two models, the cynomolgus macaque used was included as repeated in the model to consider the proper variability of each animal. A first-order autoregressive covariance matrix (AR1) to take into account the intra-subject variance/covariance structure was chosen. To guarantee an overall 5% error risk, when all the time points post-vaccination were compared to the baseline, a Dunnett’s adjustment was applied.

## Data Availability

The transcriptomics data discussed in this publication have been deposited in NCBI's Gene Expression Omnibus (Edgar et al., 2002) and are accessible through GEO Series accession number GSE253538. CyTOF data are available on the Flow Repository site at http://flowrepository.org/id/FR-FCM-Z77R for human samples and at http://flowrepository.org/id/FR-FCM-Z77L for cynomolgus data.

## SUPPLEMENTAL MATERIAL

Supplemental material jvi.01516-23-s0001.docx Table S1; Fig. S1 to S11.

ASM does not own the copyrights to Supplemental Material that may be linked to, or accessed through, an article. The authors have granted ASM a non-exclusive, world-wide license to publish the Supplemental Material files. Please contact the corresponding author directly for reuse.
