# Supplementary material for: Cynomolgus macaques as a translational model of human immune responses to yellow fever 17D vaccination
Source: J Virol. 2024 Apr 3;98(5):e01516-23. doi: 10.1128/jvi.01516-23 (PMC11092345; doi:10.1128/jvi.01516-23)
Supplement: Supplemental material — Table S1; Fig. S1 to S11. [file jvi.01516-23-s0001.docx]

**Supplementary Materials**

Cynomolgus macaques as a translational model of human immune responses to yellow fever 17D vaccination

Nathalie Mantel^a#^, Fabienne Piras-Douce^a^, Emilie Chautard^a^, Ernesto Marcos-Lopez^b^, Caroline L Bodinham^c^, Antonio Cosma^b^, Virginie Courtois^a^, Nina Dhooge^b^, Sylviane Gautheron^a^, Stefan HE Kaufmann^d,e^, Kathleen Pizzoferro^c^, David JM Lewis^c^, Frédéric Martinon^b^, Anke Pagnon^a^, Franck Raynal^a^, Nathalie Dereuddre-Bosquet^b^, Roger Le Grand^b^

*^a^Research and Development, Sanofi, Marcy l’Etoile, France.*

*^b^Université Paris-Saclay, INSERM, CEA, Center for Immunology of Viral, Auto-immune, Hematological and Bacterial diseases (IMVA-HB/IDMIT), Fontenay aux Roses, France.*

*^c^Surrey Clinical Research Centre, University of Surrey, Guildford, Surrey, UK.*

*^d^Max Planck Institute for Infection Biology, Berlin, Germany; Max Planck Institute for Multidisciplinary Sciences, Göttingen, Germany*

*^e^Hagler Institute for Advanced Study, Texas A&M University, College Station, Texas, USA.*

**Results: Vaccine induced changes in myeloid and lymphoid leukocyte (phenotypic analysis of SPADE clusters)**

The selection of 300 clusters resulted in the generation of many artificial subpopulations with almost identical phenotypes, a phenomenon previously described as “over-clustering” (Saeys et al. 2016, Palgen et al. 2018). However, we consider that “over-clustering” is less misleading than “under-clustering” in which two or more different cell populations may be present in one cluster. Four independent heat maps (granulocytes, monocytes and DCs, T and NK cells, and B cells) were generated to stratify the eight cell populations into phenotypic families (**Figure 3** and **4 [see main paper]**). This approach helps to prevent misinterpretations of results due to “over-clustering” by grouping very similar clusters into the same phenotypic family.

Human monocytes were grouped into 52 clusters and 9 phenotypic families (G–O) and DCs in 17 clusters and 6 phenotypic families (A–F) (**Figure 3A [see main paper]**). Two clusters of plasmacytoid dendritic cell (pDCs) identified were grouped in family B. For cynomolgus macaques, monocytes were grouped into 53 clusters and 12 phenotypic families (A–L) and DCs into 35 clusters and 8 phenotypic families (M–T), with two clusters of pDC identified grouped in family S.

Human granulocytes were grouped into 102 clusters and 25 phenotypic families (A–Y), highlighting the phenotypic heterogeneity of this cell population (**Figure 4A [see main paper]**). Among granulocytes, basophils were grouped into three clusters and one phenotypic family (L). Cynomolgus macaque granulocytes were grouped into only 47 clusters and 15 phenotypic families (A–O); five basophil clusters were distributed into two phenotypic families (A and B).

Although the panels were not designed to deeply characterize lymphocytes, human B cells were grouped into 23 clusters and 10 phenotypic families (A–J), whereas cynomolgus macaque B cells were grouped into 56 clusters and 7 phenotypic families (A-G) (**Figure 3B [see main paper]**). Finally, human T cells were grouped into 86 clusters and 18 phenotypic families (A–P, S and T) and NK cells grouped into 13 clusters and two families (Q and R) (**Figure 4B [see main manuscript]**). In comparison, cynomolgus macaque T cells were grouped into 54 clusters and 15 phenotypic families (F–T) and NK cells into 22 clusters and five phenotypic families (A–E).

**References**

Palgen JL, Tchitchek N, Elhmouzi-Younes J, Delandre S, Namet I, Rosenbaum P, Dereuddre-Bosquet N, Martinon F, Cosma A, Levy Y, Le Grand R, Beignon AS (2018). "Prime and Boost Vaccination Elicit a Distinct Innate Myeloid Cell Immune Response." Sci Rep **8**(1): 3087.

Saeys Y, Van Gassen S, Lambrecht BN (2016). "Computational flow cytometry: helping to make sense of high-dimensional immunology data." Nat Rev Immunol **16**(7): 449-462.

**Table S1.** P-values of significant changes of phenotypic families presented in Figure 5B

| **Species** | **Cell subsets** | **Phenotypic families** | **Time points** | **p-values** |
| --- | --- | --- | --- | --- |
| Cynomolgus macaques | Monocytes & DCs | A | D03 | 0.005 |
| Cynomolgus macaques | Monocytes & DCs | B | D03 | 0.010 |
| Cynomolgus macaques | Monocytes & DCs | C | D03 | 0.009 |
| Cynomolgus macaques | Monocytes & DCs | A | D07 | 0.008 |
| Cynomolgus macaques | Monocytes & DCs | D | D07 | 0.003 |
| Cynomolgus macaques | Monocytes & DCs | D | D14 | 0.007 |
| Cynomolgus macaques | Monocytes & DCs | M | D14 | 0.009 |
| Cynomolgus macaques | Monocytes & DCs | A | D28 | <0.001 |
| Cynomolgus macaques | Monocytes & DCs | C | D28 | 0.010 |
| Cynomolgus macaques | Monocytes & DCs | D | D28 | 0.002 |
| Cynomolgus macaques | Monocytes & DCs | K | D28 | 0.005 |
| Cynomolgus macaques | B cells | G | D01 | 0.001 |
| Cynomolgus macaques | B cells | F | D03 | 0.001 |
| Cynomolgus macaques | B cells | G | D03 | 0.002 |
| Cynomolgus macaques | B cells | F | D07 | 0.003 |
| Cynomolgus macaques | B cells | G | D07 | 0.005 |
| Cynomolgus macaques | B cells | F | D14 | 0.003 |
| Cynomolgus macaques | B cells | G | D14 | 0.002 |
| Cynomolgus macaques | B cells | F | D28 | 0.001 |
| Cynomolgus macaques | B cells | G | D28 | <0.001 |
| Cynomolgus macaques | Granulocytes | F | D01 | 0.002 |
| Cynomolgus macaques | Granulocytes | H | D01 | 0.007 |
| Cynomolgus macaques | Granulocytes | J | D01 | 0.006 |
| Cynomolgus macaques | Granulocytes | F | D03 | 0.003 |
| Cynomolgus macaques | Granulocytes | H | D03 | <0.001 |
| Cynomolgus macaques | Granulocytes | J | D03 | 0.002 |
| Cynomolgus macaques | Granulocytes | F | D07 | 0.001 |
| Cynomolgus macaques | Granulocytes | J | D07 | 0.0001 |
| Cynomolgus macaques | Granulocytes | F | D14 | 0.005 |
| Cynomolgus macaques | Granulocytes | H | D14 | 0.007 |
| Cynomolgus macaques | Granulocytes | J | D14 | 0.001 |
| Cynomolgus macaques | Granulocytes | F | D28 | <0.001 |
| Cynomolgus macaques | Granulocytes | J | D28 | 0.002 |
| Cynomolgus macaques | T cells & NK | D | D01 | 0.007 |
| Cynomolgus macaques | T cells & NK | F | D01 | 0.006 |
| Cynomolgus macaques | T cells & NK | H | D01 | 0.004 |
| Cynomolgus macaques | T cells & NK | K | D01 | 0.001 |
| Cynomolgus macaques | T cells & NK | B | D03 | 0.003 |
| Cynomolgus macaques | T cells & NK | F | D03 | 0.001 |
| Cynomolgus macaques | T cells & NK | H | D03 | <0.001 |
| Cynomolgus macaques | T cells & NK | I | D03 | 0.005 |
| Cynomolgus macaques | T cells & NK | D | D07 | 0.002 |
| Cynomolgus macaques | T cells & NK | H | D07 | 0.009 |
| Cynomolgus macaques | T cells & NK | K | D07 | 0.001 |
| Cynomolgus macaques | T cells & NK | L | D07 | 0.003 |
| Cynomolgus macaques | T cells & NK | D | D14 | 0.009 |
| Cynomolgus macaques | T cells & NK | H | D14 | 0.007 |
| Cynomolgus macaques | T cells & NK | R | D14 | 0.004 |
| Cynomolgus macaques | T cells & NK | D | D28 | 0.005 |
| Cynomolgus macaques | T cells & NK | H | D28 | 0.007 |
| Cynomolgus macaques | T cells & NK | K | D28 | 0.001 |
| Cynomolgus macaques | T cells & NK | L | D28 | 0.002 |
| Human | Monocytes & DCs | F | D07 | 0.001 |
| Human | Monocytes & DCs | A | D14 | 0.003 |
| Human | Monocytes & DCs | B | D14 | <0.001 |
| Human | Monocytes & DCs | E | D14 | 0.004 |
| Human | Granulocytes | O | D07 | 0.006 |
| Human | Granulocytes | O | D14 | 0.006 |
| Human | Granulocytes | T | D14 | 0.004 |
| Human | T cells & NK | B | D07 | 0.004 |
| Human | T cells & NK | J | D14 | 0.001 |
| Human | T cells & NK | B | D28 | 0.002 |

**Figure S1.** Changes in bodyweight and rectal temperatures in cynomolgus macaques (n=12) following vaccination with YF-17D. Individual data presented. The vertical dotted line indicates day of vaccination

**Figure S2.** Hematology in whole blood from YF-17D vaccinated cynomolgus macaques (red) versus adult human participants (blue). Mean value (Log; ratio versus baseline) ± 95% confidence intervals at each time point

| White blood cells  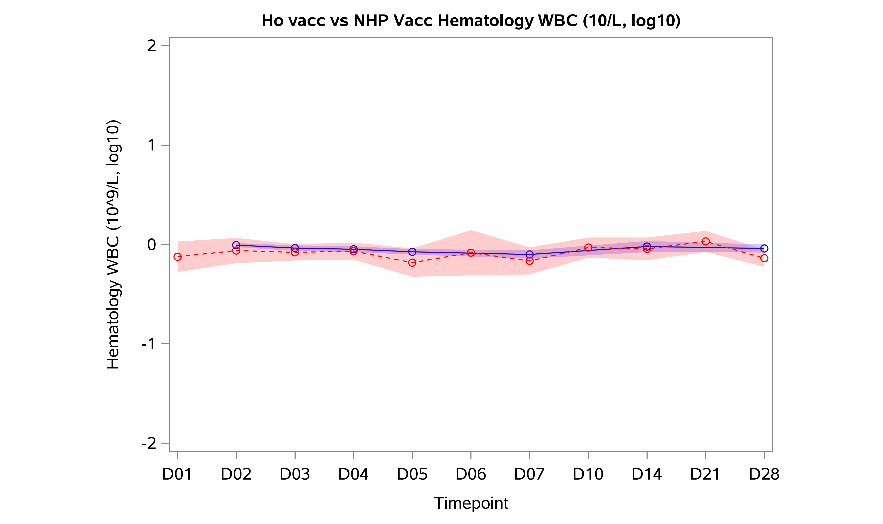 | Neutrophils  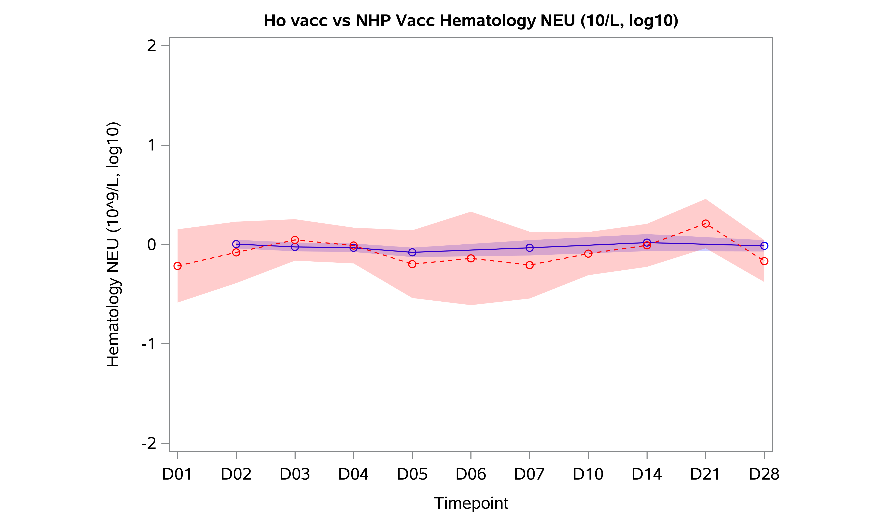 | Lymphocytes  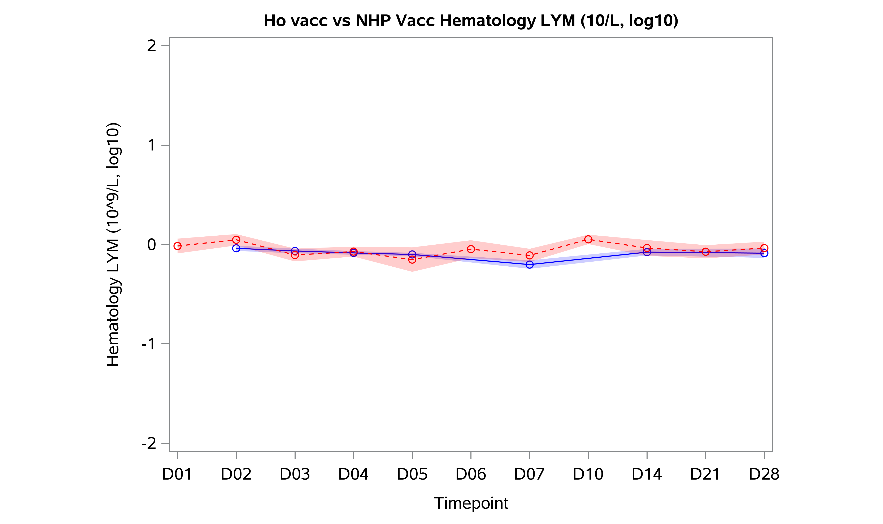 |
| --- | --- | --- |
| Monocytes  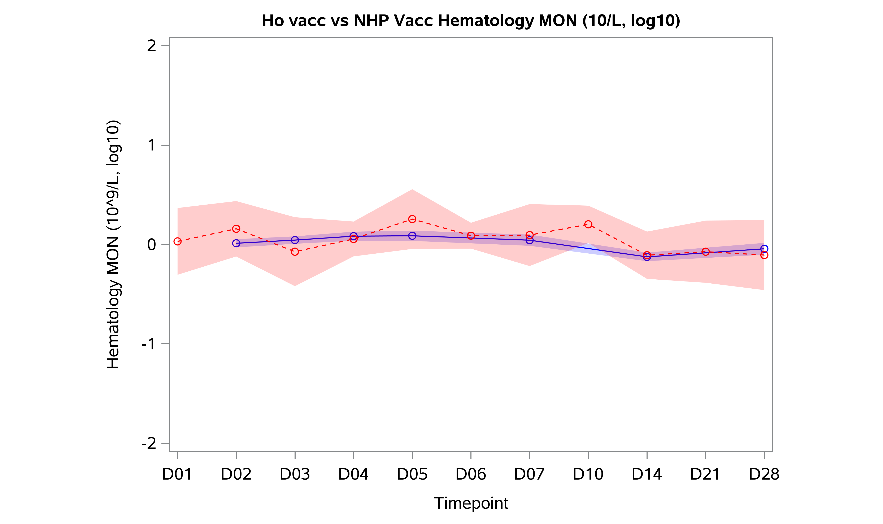 | Eosinophils  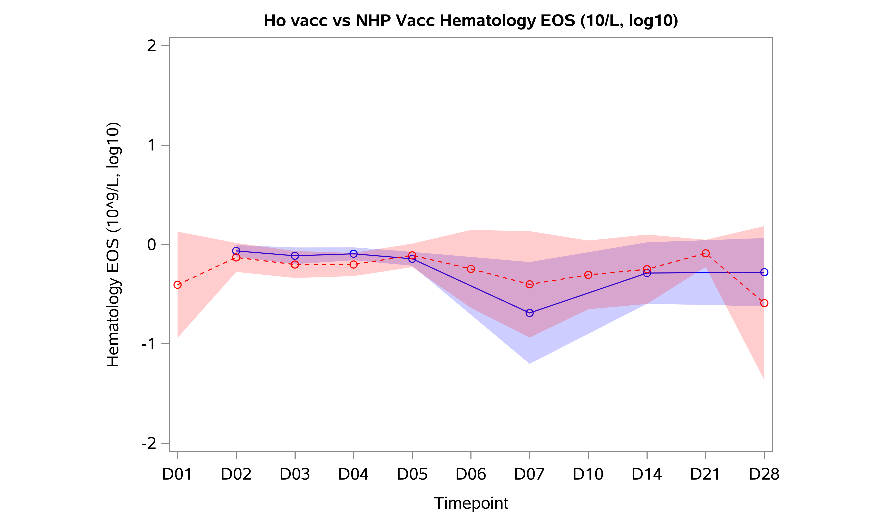 | Basophils  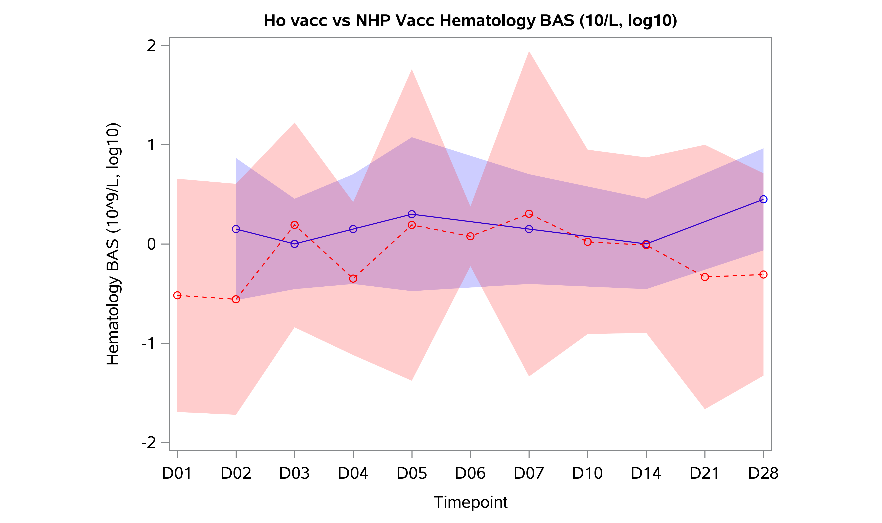 |
| Red blood cells  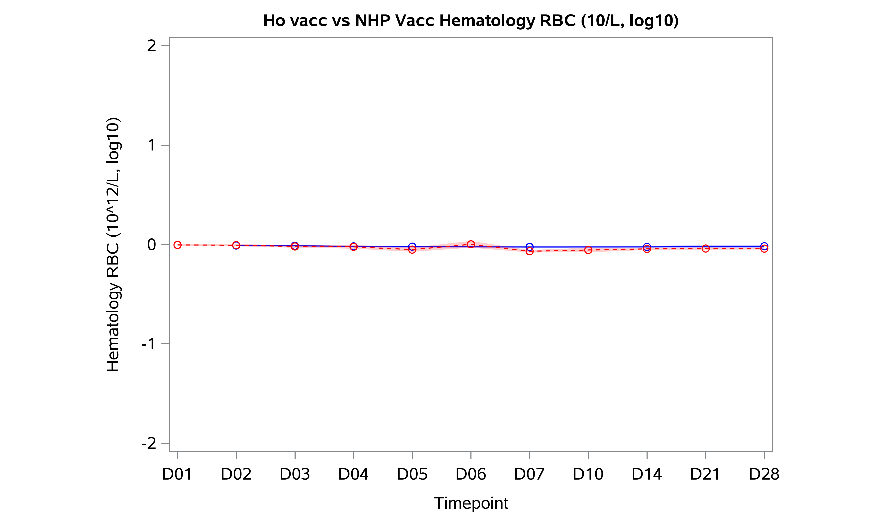 | Hemoglobulin  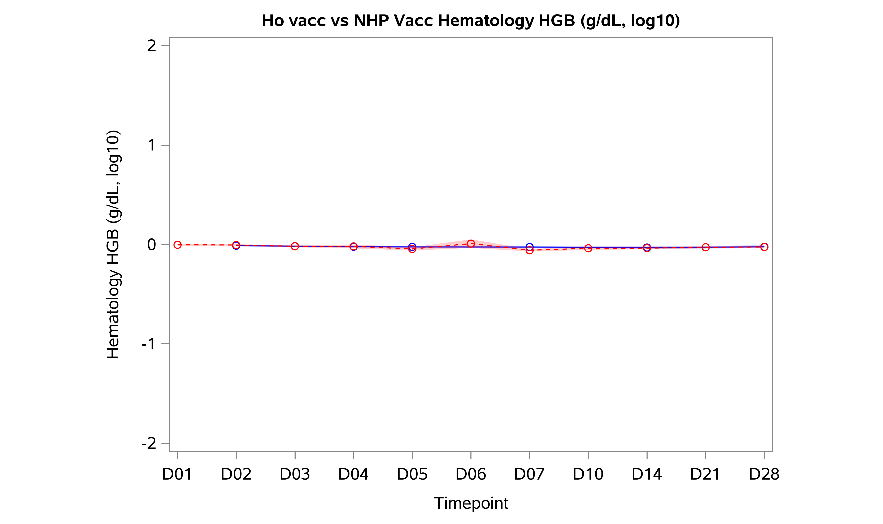 | Hematocrit  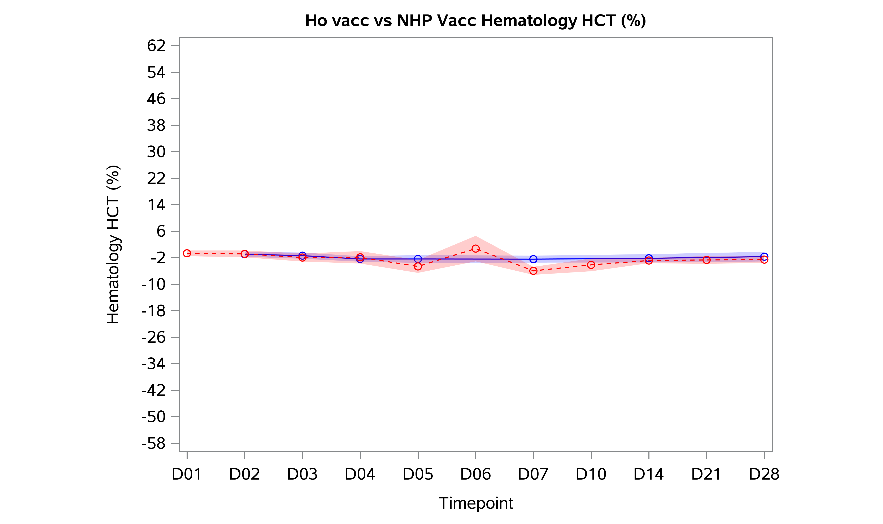 |
| Mean corpuscular Volume  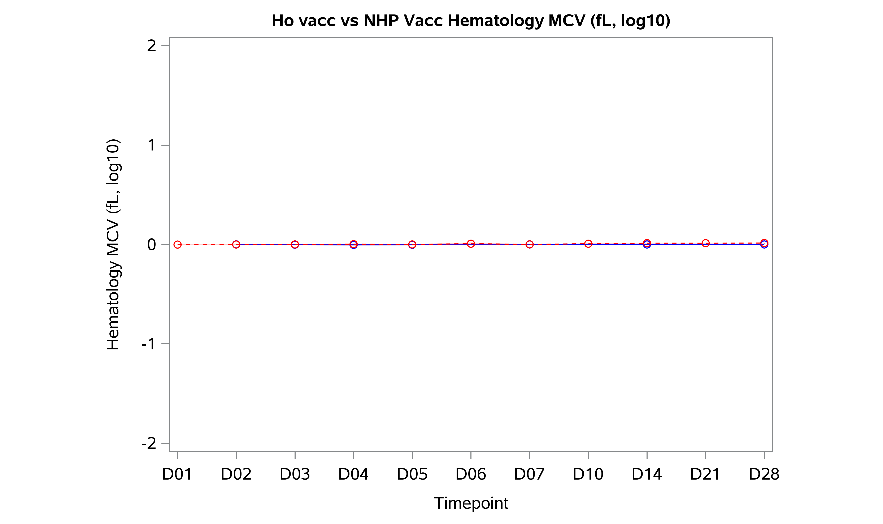 | Mean corpuscular hemoglobin  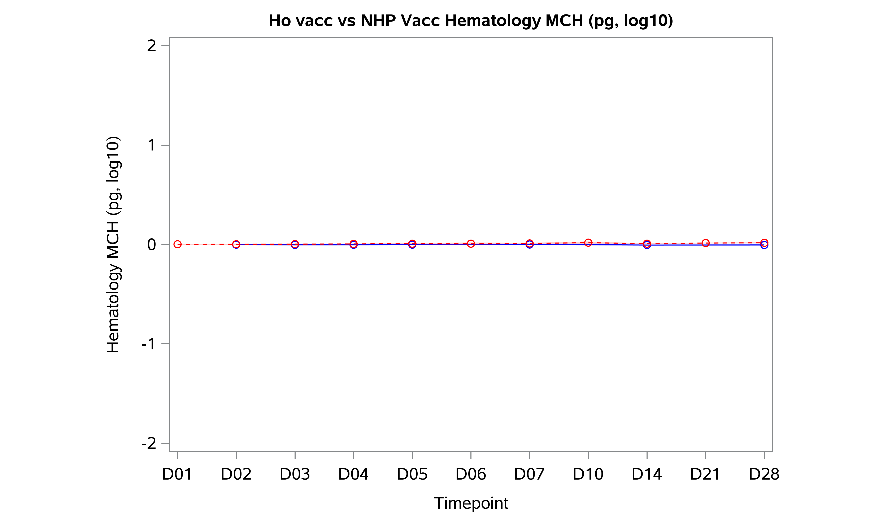 | Mean corpuscular hemoglobin concentration  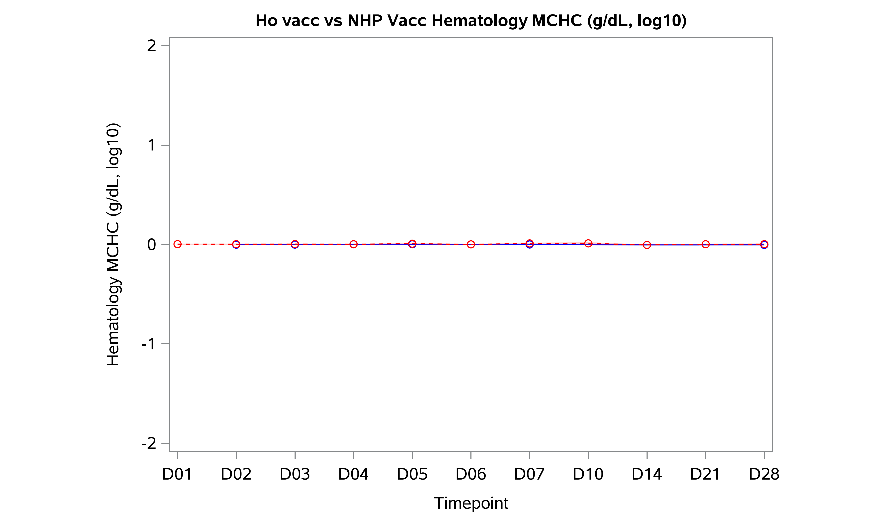 |
| Platelets  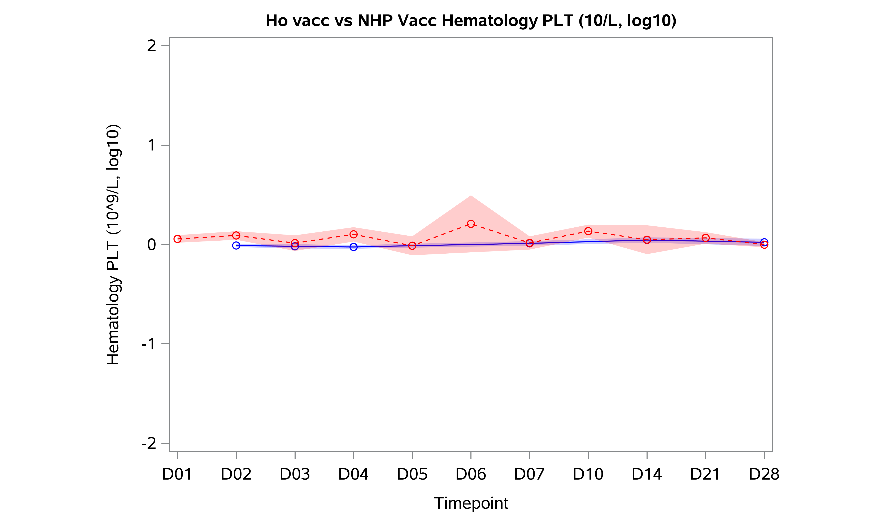 |  |  |

**Figure S3.** Blood biochemistry in serum from YF-17D vaccinated cynomolgus macaques (red) versus adult human participants (blue). Mean value (Log; ratio versus baseline) ± 95% confidence intervals at each time point

| Albumin  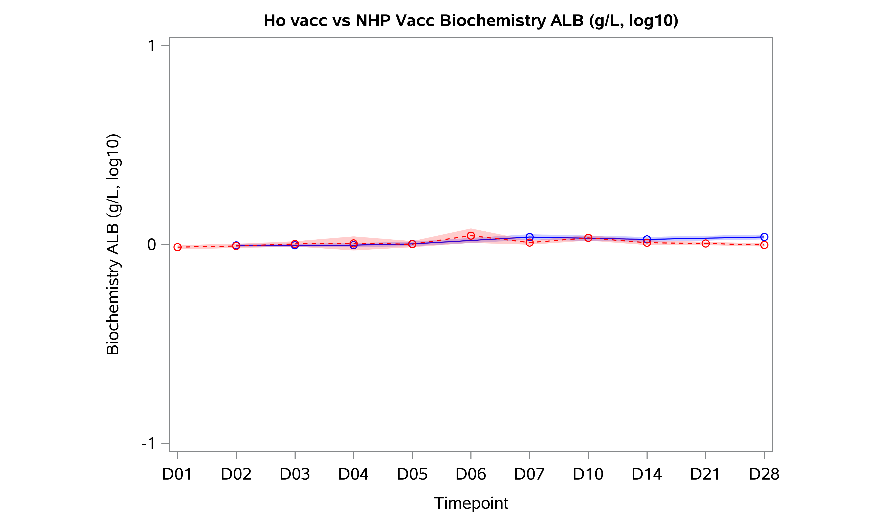 | Alanine aminotransferase  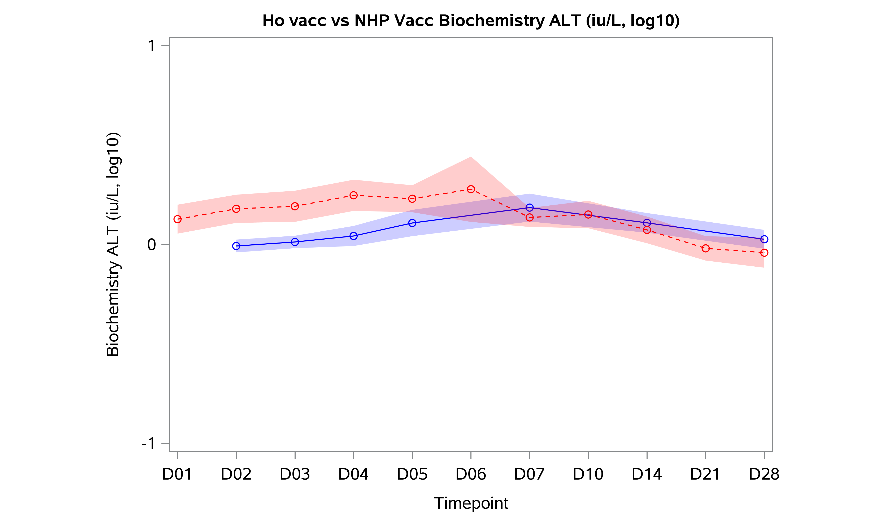 | Total bilirubin  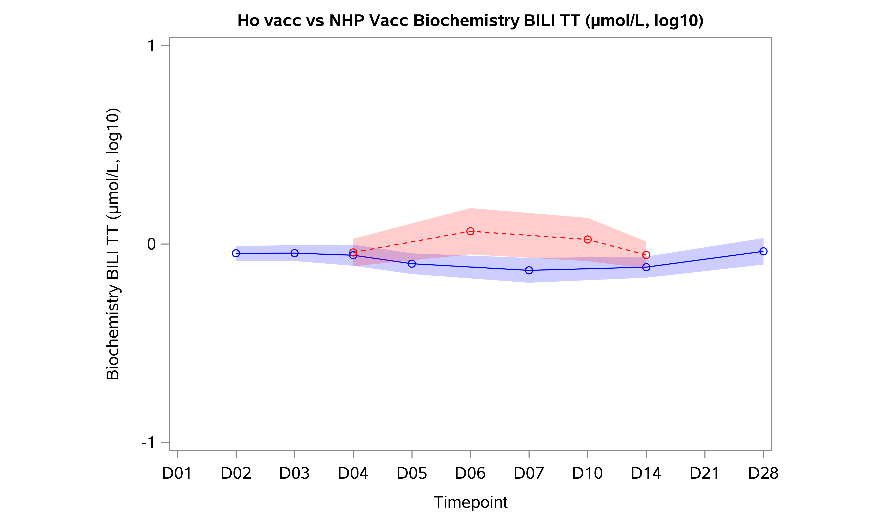 |
| --- | --- | --- |
| Creatinine  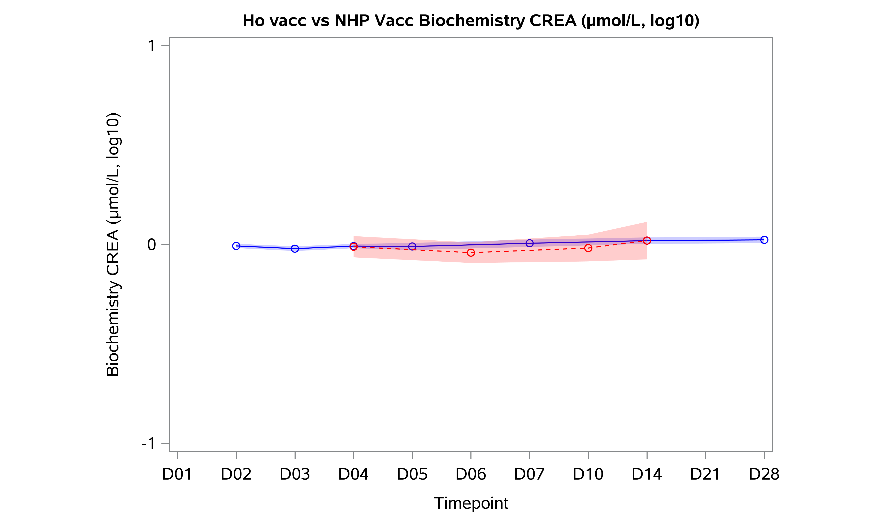 | Gamma-glutamyl transferase  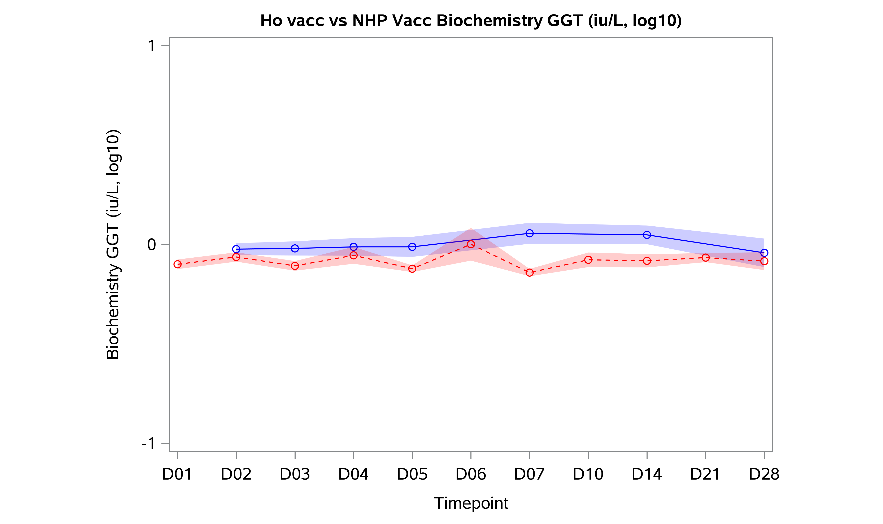 | Alkaline phosphatase  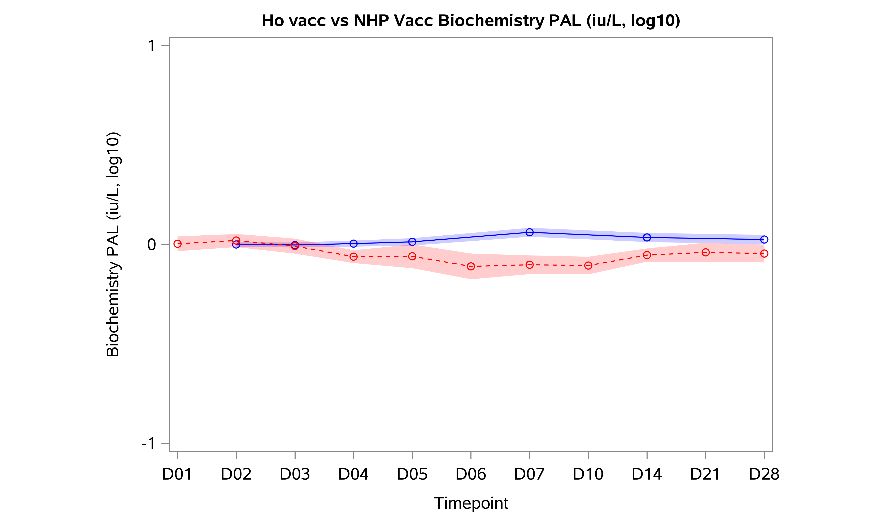 |
| Urea  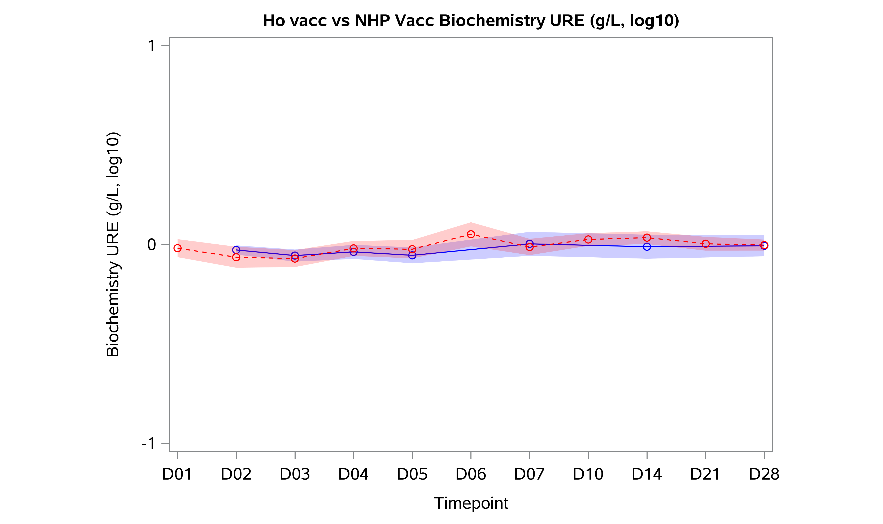 | C-reactive protein  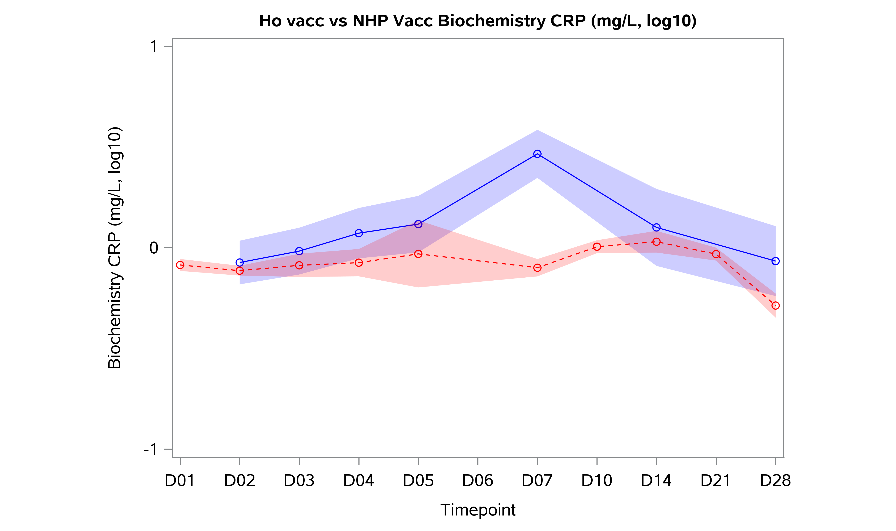 |  |
|  |  |  |

**Figure S4.** Summary of cytokine, chemokines and growth factor response profile in cynomolgus macaques (red) and human participants (blue) following YF-17D vaccination. Data shown as mean value (Log; ratio versus baseline) ±95% confidence intervals at each time point.


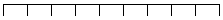

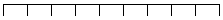

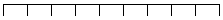

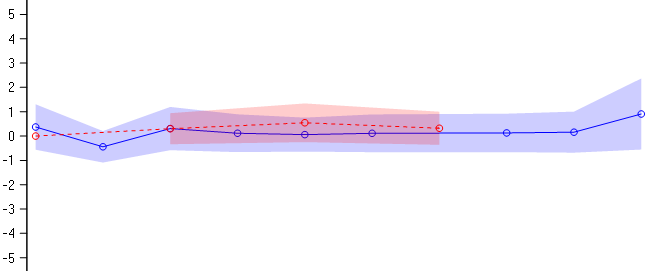


**IFNα2**

Cytokines (Log ratio vs baseline)

Days post-Vaccination


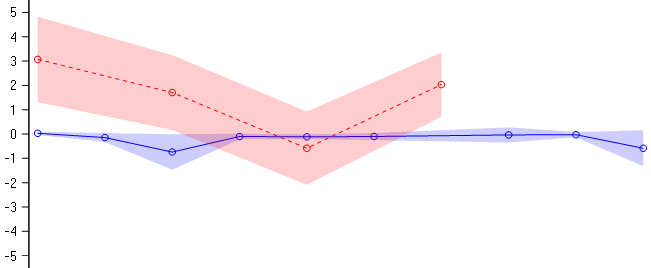


**VEGF-A**


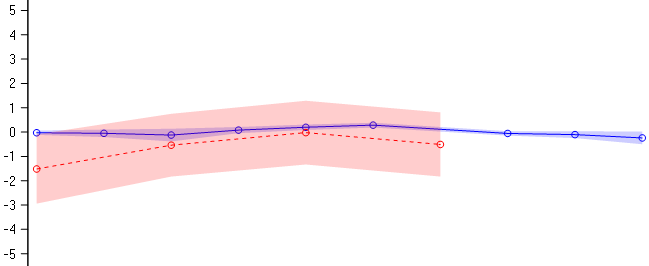


**IP-10**


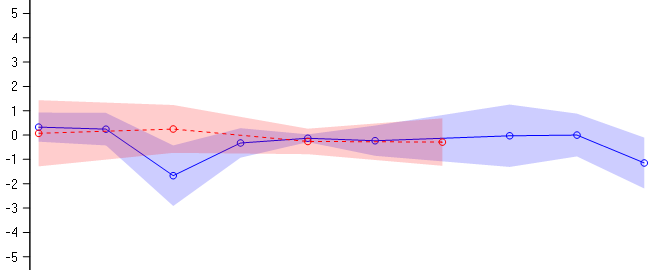


**MIP1α**


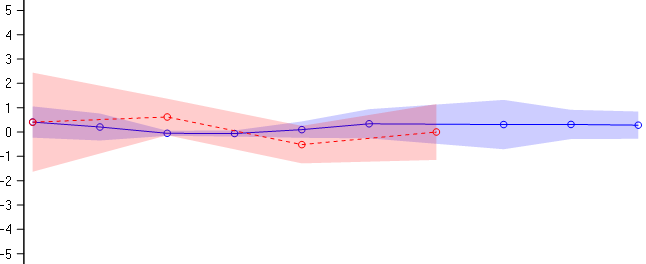


**TNF-α**


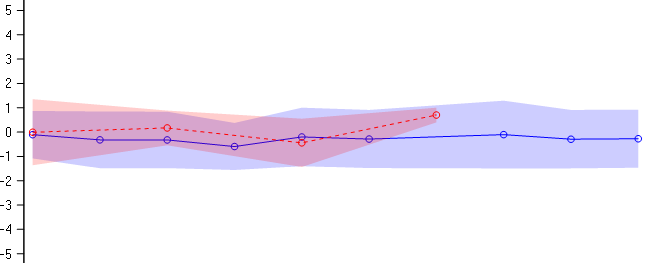


**IL-8**


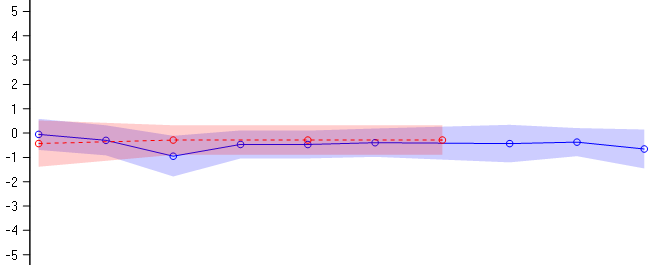


**GM-CSF**


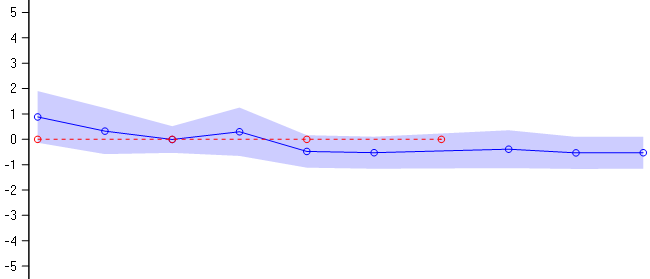


**IL-6**


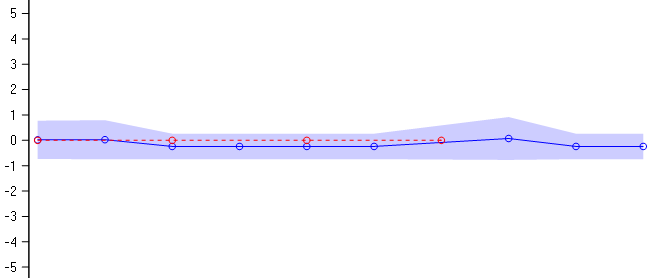


**IL-2**


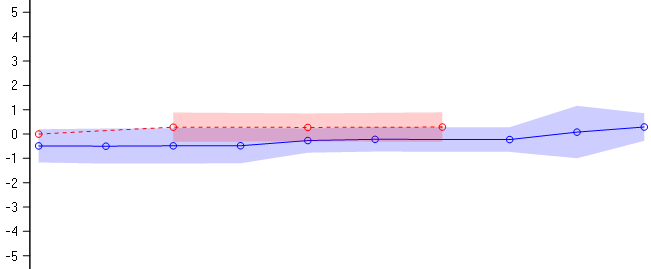


**IL-12p70**


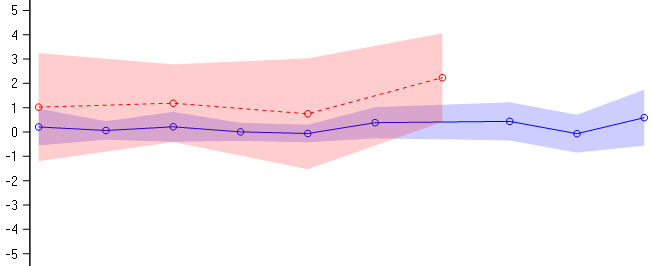


**IL1-RA**


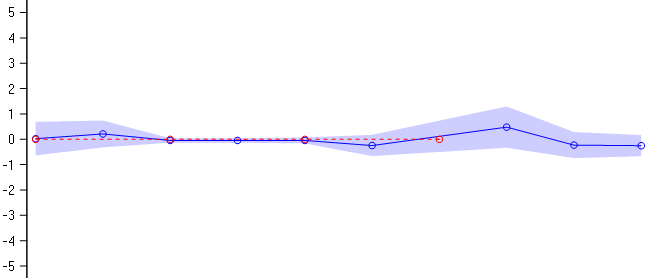


**IL-5**


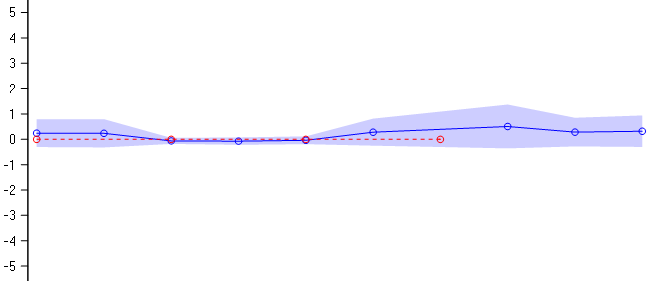


**IL-10**


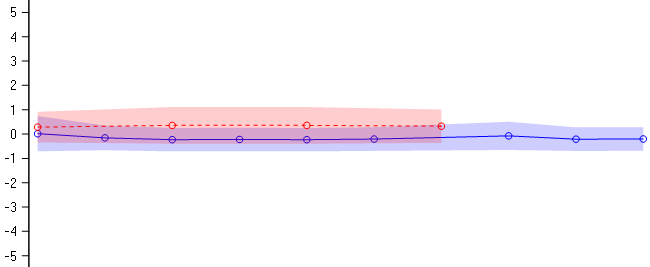


**IFNγ**


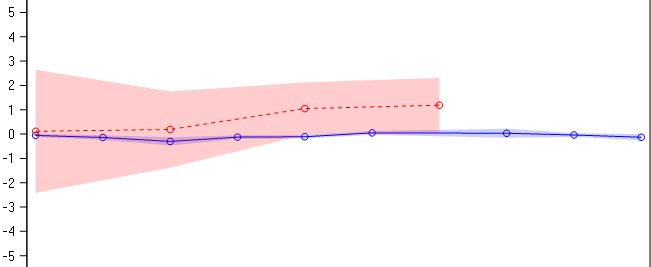


**MCP1**

**Figure S5.** Anti-YF NS1 IgG titers in serum of vaccinated cynomolgus macaques (N=18) and adult human participants (N=20)

Dashed line = Limit of detection (LOD); bar represents the mean and the dots individual data for cynomolgus macaques or human participants

**Figure S6.** Heatmap showing the gene expressed in adult human participants (placebo effect subtracted)

**
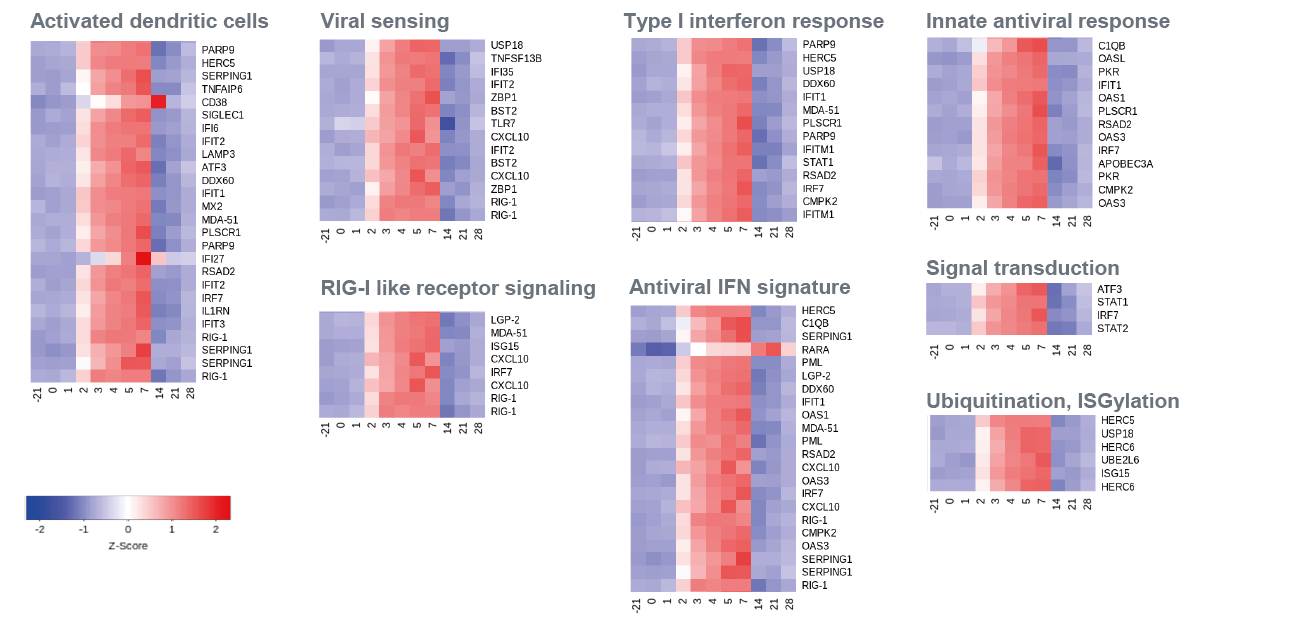
**

**Figure S7.** “Martinelli immature neutrophil up” (M14418, in the C2 curated gene sets collection, subcollection GCP: chemical and genetic perturbations gene sets)


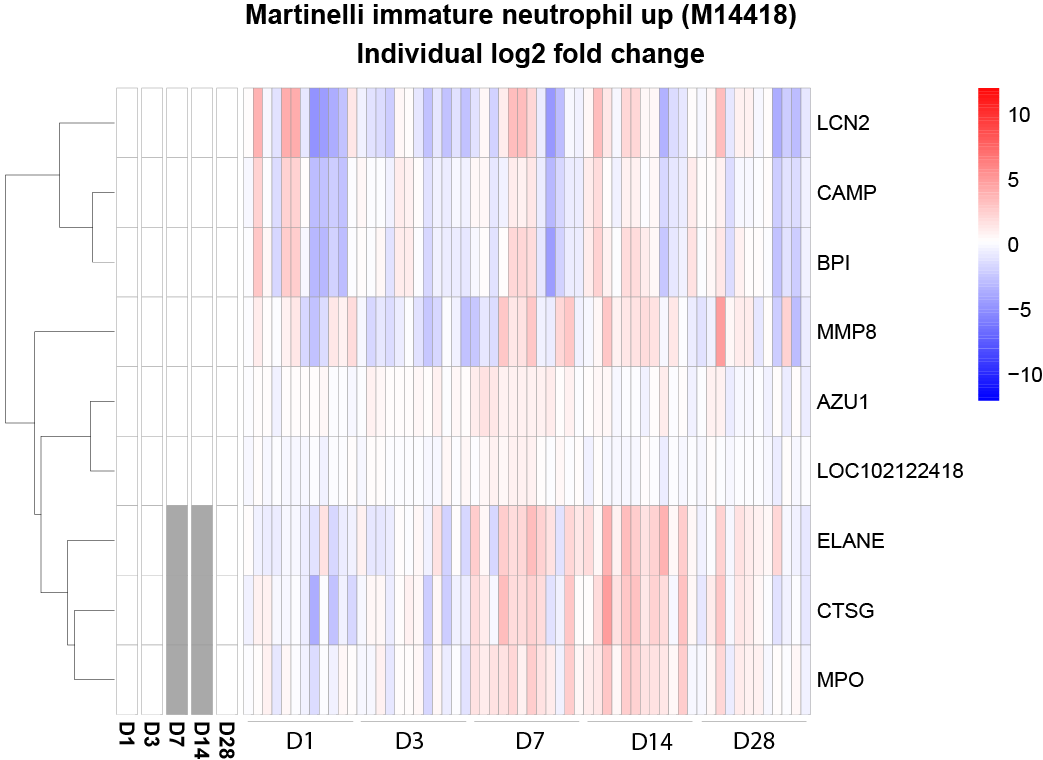


**Figure S8.** Tmod showing the YF-17D and placebo groups analyzed separately (erythrocyte production pathway) in human participants

**
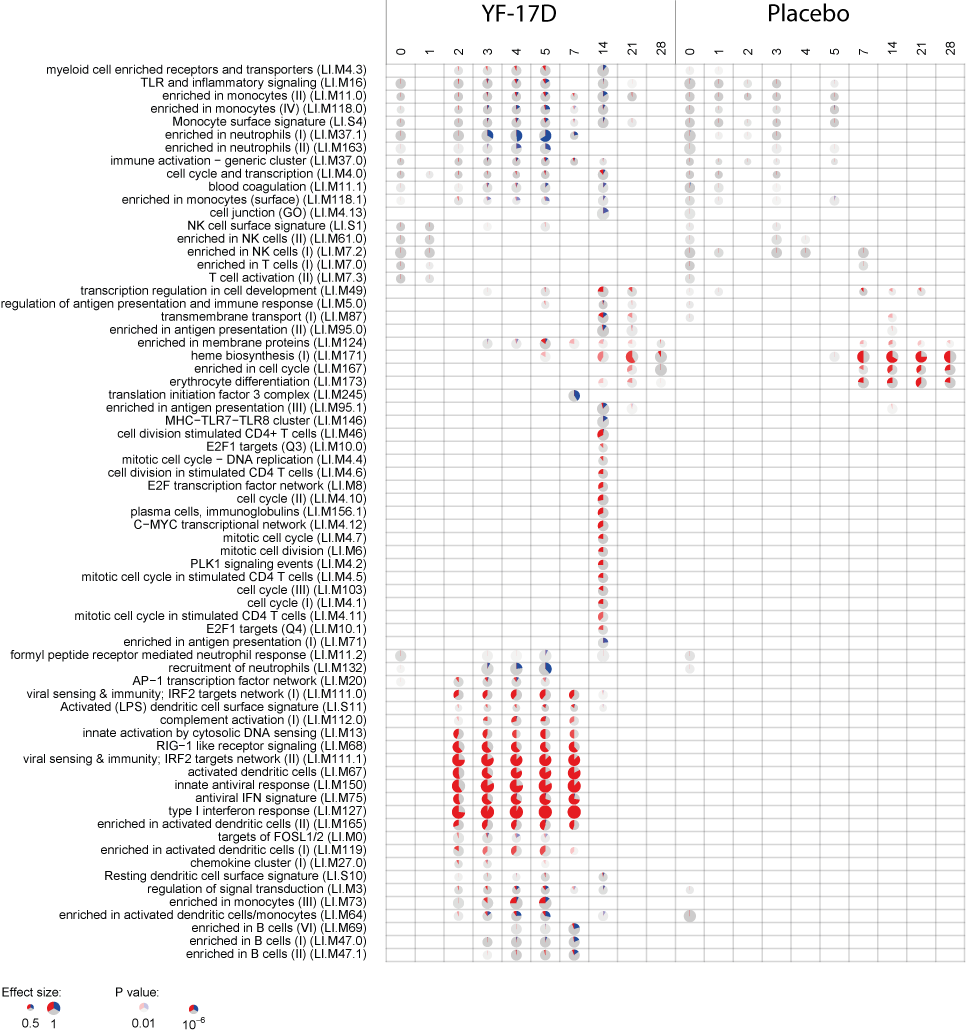
**

**Figure S9.** Expression profiles of selected gene parts of stimulated CD4 T cell and cell cycle modules in cynomolgus macaques at D7 and adult human participants at D14

**Figure S10.** Tmod from previously described publications of transcriptomics data applied to human clinical trials (Gaucher et al 2008; Querec et al 2009; Hou et al 2017)

Gaucher D, Therrien R, Kettaf N, Angermann BR, Boucher G, Filali-Mouhim A, Moser JM, Mehta RS, Drake DR, 3rd, Castro E et al: Yellow fever vaccine induces integrated multilineage and polyfunctional immune responses. J Exp Med 2008, 205(13):3119-3131.

Querec TD, Akondy RS, Lee EK, Cao W, Nakaya HI, Teuwen D, Pirani A, Gernert K, Deng J, Marzolf B et al: Systems biology approach predicts immunogenicity of the yellow fever vaccine in humans. Nat Immunol 2009, 10(1):116-125.

Hou J, Wang S, Jia M, Li D, Liu Y, Li Z, Zhu H, Xu H, Sun M, Lu L et al: A Systems Vaccinology Approach Reveals Temporal Transcriptomic Changes of Immune Responses to the Yellow Fever 17D Vaccine. J Immunol 2017, 199(4):1476-1489.

**Figure S11.** Mass cytometry. Fixed leukocytes were stained with a panel of antibodies designed to analyze whole blood cells by mass cytometry. Targeted markers and their associated biological functions are indicated for (**A**) humans and (**B**) macaques. The 17 markers that were used to cluster the cells using SPADE are shown in blue. Non-common markers between humans and macaques are shown in grey. Markers in red represent common but not clustering markers. (**C**) Events generated from human and macaque samples were manually gated to exclude the EQTM Four-Element Calibration Beads, select singlets and gate out nonspecific background likely generated by metal conjugated antibody binding eosinophils (see Materials and Methods). (D) The steps of the mass cytometry data analysis are displayed. As the first analysis step, single cells from FCS files were grouped into clusters sharing similar phenotype using the SPADE algorithm. Clusters were annotated on the resulting SPADE tree based on the expression of a set of 16 markers. As the second analysis step, clusters sharing the same categories of marker expression were regrouped into phenotypic families. (E) Mass cytometry data were analyzed using SPADE. The topology of the SPADE tree is shown. This tree was built using all samples (all subjects and all time points). Only the topology of the tree is displayed where each node corresponds to a cell cluster. Clusters with similar phenotypes are linked using a minimal spanning tree approach.

**
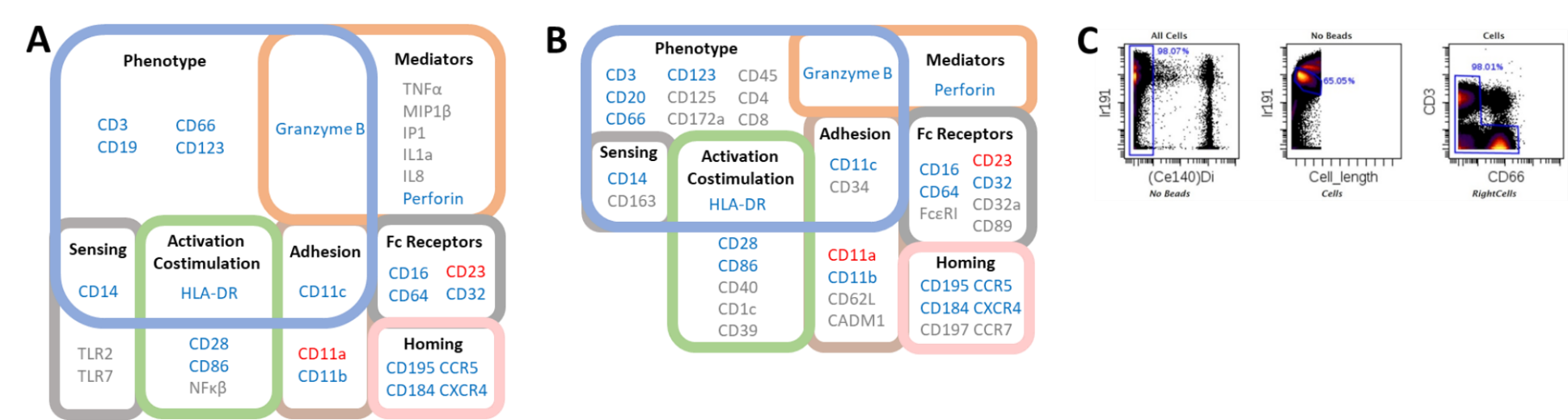
**

**
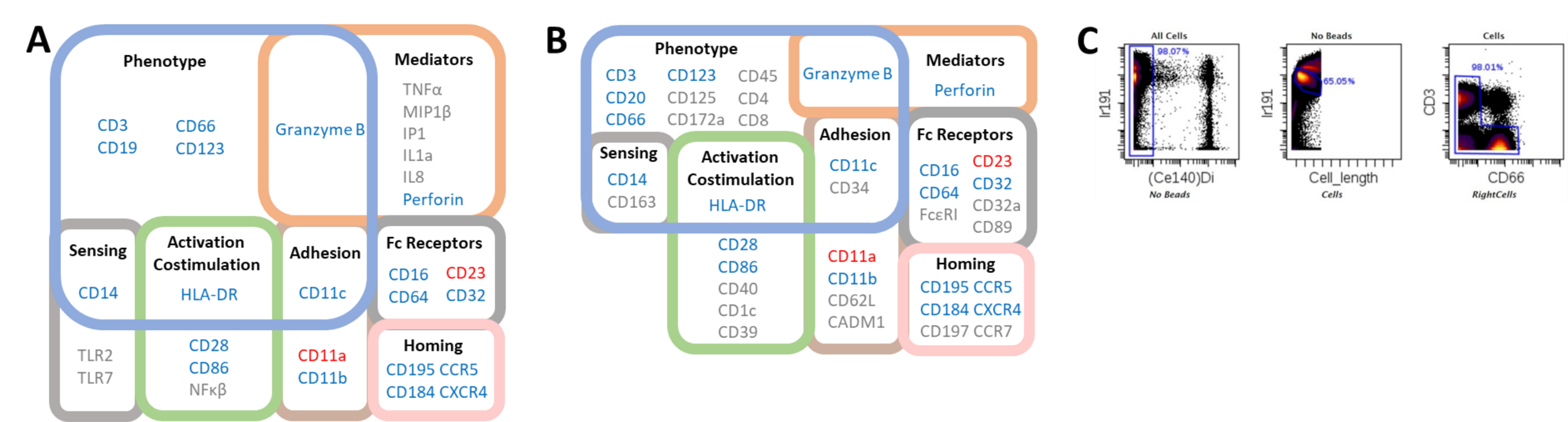
**
